# The effect of transcranial direct current stimulation on frontal alpha asymmetry and visuospatial attention in food-reward contexts: a triple-blind randomized sham-controlled study

**DOI:** 10.1186/s40359-025-02972-x

**Published:** 2025-07-01

**Authors:** Atakan M. Akil, Renáta Cserjési, Dezső Németh, Tamás Nagy, Zsolt Demetrovics, H. N. Alexander Logemann

**Affiliations:** 1 Institute of Psychology, ELTE Eötvös Loránd University, Izabella utca 46, Budapest 1064, Hungary; 2 Institute of Psychology, Faculty of Humanities and Social Sciences, University of Pécs, Ifjúság útja 6, Pécs H-7624, Hungary; 3 INSERM, Université Claude Bernard Lyon 1, CNRS, Centre de Recherche en Neurosciences de Lyon CRNL U1028 UMR5292, Bron, France; 4 NAP Research Group, Institute of Psychology, Eötvös Loránd University & Institute of Cognitive Neuroscience and Psychology, HUN-REN Research Centre for Natural Sciences, Budapest, Hungary; 5 Gran Canaria Cognitive Research Center, University of Atlántico Medio, Las Palmas de Gran Canaria, Spain; 6 Institute for Mental Health and Wellbeing, College of Education, Psychology and Social Work, Flinders University, Bedford Park, South Australia Australia; 7 Centre of Excellence in Responsible Gaming, University of Gibraltar, Gibraltar, Gibraltar; 8 Department of Clinical, Neuro and Developmental Psychology, Vrije Universiteit Amsterdam, De Boelelaan, Amsterdam, 1105 1081 HV Netherlands

**Keywords:** Attentional bias, Attentional disengagement, Frontal alpha asymmetry, Inhibitory control, Transcranial direct current stimulation, Visuospatial attention

## Abstract

**Background:**

Research indicates a connection between right frontal brain activity dominance and the inhibitory system. However, the underlying brain mechanism regarding attentional control remains unclear. In this preregistered experiment, we explored the association between frontal alpha asymmetry (FAA) — which is an electrophysiological measure of the difference in alpha power between the left and right dorsolateral prefrontal cortex — and the behavioral and brain activity components of attentional bias and disengagement, as observed in a visuospatial cueing (VSC) task.

**Method:**

We used a triple-blind randomized sham-controlled design with 65 (*M*_age_ = 23.93; *SD*_age_ = 6.08; 46 female) healthy humans. Participants’ resting-state EEG was recorded to calculate FAA scores before and after 2 mA anodal transcranial direct current stimulation (tDCS) to the right frontal site for 20 min. They also completed a VSC task with neutral and intrinsic reward-associated (food) conditions.

**Results:**

Results indicated no impact of tDCS on FAA or behavioral attentional bias and disengagement. Exploratory analyses revealed tDCS-induced attentional bias for rewards, as detected in enhanced Late Directing Attention Positivity and P1 effect. However, these effects did not translate into observable behavioral changes.

**Conclusion:**

The enhanced attentional bias may result from tDCS-induced enhancement of top-down attentional orienting to spatial locations associated with reward-related stimuli, potentially mediated by noradrenergic mechanisms.

**Supplementary Information:**

The online version contains supplementary material available at 10.1186/s40359-025-02972-x.

## Background

Within the domain of visuospatial attention, attentional bias and attentional disengagement are not just essential for understanding cognitive processes, but also crucial for a healthy life, as attentional bias, especially towards mood-congruent stimuli, and challenges in attentional disengagement, particularly within the framework of inhibitory control [1], have been associated with various psychiatric disorders such as addiction [2] depression [3], and anxiety disorders [4]. When an observer anticipates a stimulus at a particular position in a visual scene, a top-down (cognitive) mechanism called attentional bias arises, which leads to an accelerated processing of the stimulus at the attended location [5]. Conversely, attentional disengagement is a stimulus-driven bottom-up (sensory) mechanism. When the observer has to focus on a more important stimulus at the unattended site, it simplifies the decoupling of attention from the attended location, which indicates a conceptual overlap with inhibitory control [1, 6, 7]. Although previous research has suggested a connection between inhibitory motivations and the dominance of the right dorsolateral prefrontal cortex (DLPFC) (at least in part), in contrast to the left, which is presumably linked with approach motivation [8, 9], the precise relationship between asymmetric frontal brain activity and attentional control remains unclear. In the current study, we explored the association between frontal alpha asymmetry (FAA), an electrophysiological measure indicating asymmetric frontal cortical activity [10] between the left and right DLPFC [11], and behavioral and brain activity indices of attentional bias and attentional disengagement in food reward contexts.

FAA can be assessed via electroencephalogram (EEG). Evidence shows that alpha band activity (8–13 Hz) decreases during periods of heightened cortical processing [12]. A common measure of FAA is derived by subtracting the natural log of left hemisphere alpha power from the natural log of right hemisphere alpha power (ln[right alpha]—ln[left alpha]). This yields a scale where positive (higher) values indicate relatively greater left frontal activity and negative (lower) values indicate greater right frontal activity [12].

While FAA can reflect broader hemispheric activation related to motivational and emotional states, and in approach and avoidance tendencies, event-related potentials (ERPs) can provide insights into the timing and neural mechanisms of specific events. Specifically, when an event occurs, large groups of neurons are activated synchronously, resulting in postsynaptic potentials that can be detected by EEG at specific times when time-locked to the event [13]. Therefore, EEG allows us to differentiate between brain activity associated with attentional orienting (cue-related) and activity related to its associated attentional bias (target-related) [14–16].

We focused on sequential ERP activity based on previous studies. Specifically, for attentional orienting, the sequence of ERPs consists of the Early Directing Attention Negativity (EDAN) and the Late Directing Attention Positivity (LDAP) indices of parietal activity, associated with attentional orienting and bias [16]. Attentional bias was identified as the modulation of target-induced parietal P1 and N1 peaks by validity. More specifically, P1 and N1 are increased for validly cued targets compared to invalidly cued targets [14, 15]. Similarly, attentional disengagement is thought to be reflected in the modulation of the Late Positive Deflection (LPD) [14, 15] in the areas that drive inhibitory control, including the right inferior Frontal Gyrus (rIFG) and temporal parietal junction [17], and it is larger to invalidly cued targets than validly cued targets. Previous studies show that stop signals (assessed by the Stop Signal Task (SST)) evoke a positivity at around 300 ms (P300) that is modulated by successful inhibition [18]. Hence, it was argued that the LPD consists of P300 [14]: It is also enhanced when a specific stimulus requires attentional disengagement. Nevertheless, the extent to which these specific components are influenced by changes in FAA remains an open question.

Both brain and behavioral indices of attentional bias and attentional disengagement can be assessed through a visuospatial cueing (VSC) task employing the Posner paradigm [1]. In this computer-based task, a central cue, such as an arrow, anticipates the location of a stimulus presented in either the left or right hemifield. However, some trials feature incorrect cues, misdirecting the expected stimulus position (e.g., the central cue pointing to the left hemifield while the target appears in the right hemifield). Individuals are required to provide a behavioral response related to specific stimulus features (e.g., size, color, or orientation) upon stimulus presentation with a key press. A critical behavioral outcome is the difference in reaction times (RTs) between validly cued and invalidly cued targets, known as the validity effect, thought to reflect both attentional bias and attentional disengagement. Notably, cues associated with reward trigger approach motivation, manifested by left-over-right frontal brain activity, while cues indicating threat activate inhibitory motivation, represented by right-over-left frontal brain activity [9, 10, 19], as discussed previously.

Prior research has suggested that the magnitude of a reward (e.g., stimuli with high or low rewarding value) impacts attentional processes. Stimuli associated with greater rewards tend to sustain attention, even when detrimental to ongoing task performance [20]. Furthermore, stimulus type also affects inhibitory processes. A key distinction has been drawn between learned rewards (e.g., money) and intrinsic rewards (e.g., palatable, high-fat/sugar foods) [21]. Specifically, studies have shown that healthy, normal-weight individuals exhibit an implicit bias toward high-calorie foods [22, 23]. Therefore, we employed an intrinsic reward condition involving appetizing food pictures to trigger approach motivation (i.e., left DLPFC), drawing from earlier studies that highlighted differences between conditions in inhibitory and attentional processes; therefore, the effects of neuromodulation would be more apparent. There has also been discussion regarding the efficiency of the tasks utilized to measure the engagement and disengagement of attention [24]. However, such studies often intermix neutral stimuli, rewarding distractors, and target stimuli within the same blocks, making it challenging to disentangle the distinct attentional processes in various conditions. Besides, these studies primarily relied on eye-tracking. In the current study, similar to previous studies, we segregated neutral and reward blocks to circumvent this issue [21].

To explore the relationship between FAA and attentional control, we employed a non-invasive brain stimulation technique. We utilized transcranial direct current stimulation (tDCS), which applies low-level electrical currents to modulate cortical activity in targeted brain regions [25]. TDCS modulates brain activity in a polarity-dependent manner, with anodal stimulation generally enhancing neural excitability, while cathodal stimulation tends to suppress it [26]. Recent studies have shown support for the efficacy of anodal tDCS targeting the right DLPFC, with the cathode placed on the left DLPFC, in reducing cravings, diminishing the desire to binge eat, and lowering overall food intake [27–29]. Moreover, Kekic et al. have suggested that the effects of tDCS may persist for hours after administration, even following a single session [28]. Following Lapenta et al., these effects may (at least partially) be due to shifting of the balance between cue-associated approach relative to avoidance/inhibitory tendencies [29], which have been associated with asymmetry of frontal brain activity, indexed by FAA (e,g, [12]). Despite suggested effects of tDCS over the DLPFC on inhibitory control, it remains unknown, to the best of our knowledge, whether these effects can be directly attributed to tDCS induced changes in frontal EEG asymmetry, specifically resting-state FAA, and whether such alterations may affect attentional bias and attentional disengagement in intrinsic reward contexts.

Therefore, the main goal of the study was to investigate the effect of tDCS on FAA, behavioral and ERP components of attentional bias and attentional disengagement in food-reward contexts. First, we measured the resting-state FAA. Then, we utilized a VSC to assess behavioral and brain activity indices of attentional bias and attentional disengagement. Subsequently, we applied 2 milliamperes (mA) of active tDCS for 20 min for the manipulation of FAA. Finally, we measured FAA again and participants repeated the VSC task for post-intervention assessments. We hypothesized that applying active tDCS with the anode positioned over the right DLPFC and the cathode over the left DLPFC for a duration of 20 min (min) would result in lower FAA scores at frontal sites (F4-F3). This reduction would reflect increased activity in the right DLFPC relative to the left DLPFC, which would be associated with increased inhibition/avoidance and concurrent reduced attentional bias. This reduction would be evident through a decreased validity effect on response time in the VSC task, as well as reduced LDAP, N1, and P1 modulation by validity. Simultaneously, we further expected active tDCS to enhance attentional disengagement, also reflected by the reduced validity effect on response time in the VSC Task, as well as a stronger LPD response to invalid relative to validly cued targets. Lastly, it was expected that these effects would be more pronounced in the reward context relative to the neutral one.

## Methods

### Participants

The rationale for the sample size was based on a within-subject pilot study (*n* = 10), where we estimated the effect size of tDCS concerning FAA as the primary measure. Specifically, using G*Power [30, 31] with a defined power of 80 percent, alpha at .05, and an estimated FAA test–retest correlation of 0.6, we determined that a sample size of 30 in the active intervention group would allow us to detect an effect of *f* > 0.237 or η^2^p > 0.053. Eligible participants were required to be at least 18 years old and had no psychological or psychiatric disorders, frequent headaches or migraines, metallic implants, epilepsy, significant head trauma in the past, recent head trauma, pacemaker, chronic skin conditions, current drug use, or low command of English. They were also asked to avoid smoking and drinking coffee for at least two hours before the experiment. During the main study, some participants withdrew due to various reasons, such as boredom and fatigue. Consequently, a total of 65 adults (46 females) aged 18 to 58 (*M*_age_ = 23.93; *SD*_age_ = 6.08) were recruited, 33 of whom were in the active tDCS group, through social media ads and university courses. Among them, fifty-seven were right-handed. Their English level was also checked using the Common European Framework of Reference for Languages—Self-assessment Grid [32]. These standard demographic questions were implemented in Psytoolkit [33, 34]. Before participating, all individuals provided written informed consent. Each participant received either a voucher or course credit for participation. The study received approval from the Institutional Review Board at Eötvös Loránd University and was conducted following the principles of the Declaration of Helsinki and its later amendments. The participant recruitment period was between 09.05.2021 and 01.04.2022.

### Procedure

**Fig. 1:**
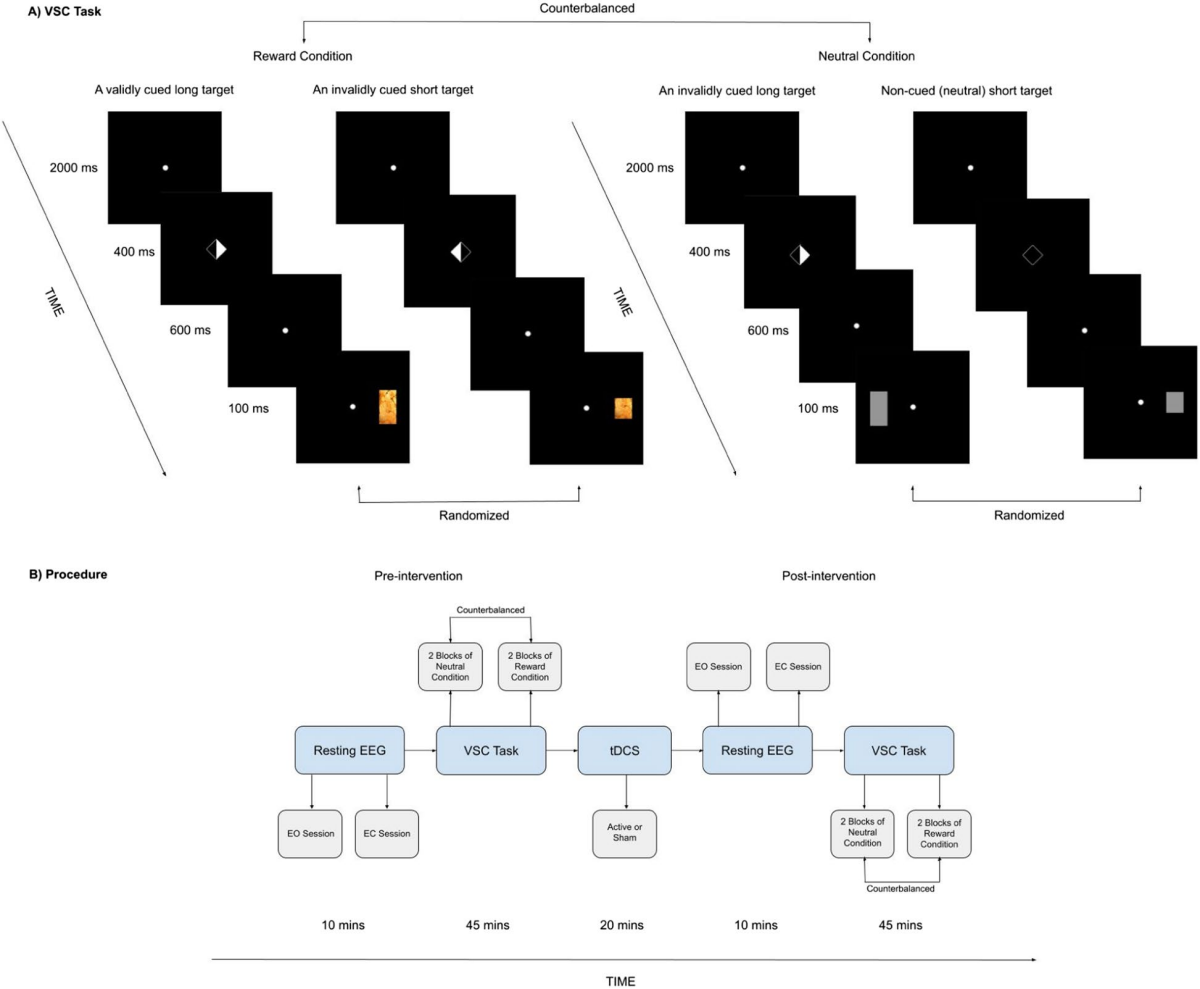
**A** This figure illustrates four example trials in the VSC task. Depending on the counterbalanced order, participants began the VSC Task in either the neutral or reward condition. The first two trials (on the left) display a validly cued long target and an invalidly cued short in the reward condition, respectively. The last two trials (on the right) represent the neutral condition. The last trial depicts a non-cued trial in which there is no cue indicating the potential location of a target, and a short neutral target is displayed on the right hemifield. The target sizes overall, and the target types in the reward condition (i.e., chocolate, cookies, nuts, and cashews) were randomized. The number of total trials was 1280 (160 trials per block, 320 trials per condition, and 640 trials per phase, i.e., either pre-intervention or post-intervention). **B** The procedure begins with collecting pre-intervention resting-state EEG data. It was collected in two sessions, one with 5 min of eyes open (EO) and another with 5 min of eyes closed (EC) conditions. Subsequently, participants performed the VSC task. Each participant received both the neutral and reward conditions in a counterbalanced order. Following that, either 2 mA active or sham tDCS over dorsolateral prefrontal cortex was administered for 20 min. In the sham condition, a brief current was applied to ensure that the sensory experience was comparable to that in the active condition. For further details regarding the intervention, please see Fig. 2. After the tDCS intervention, we repeated the same procedure for the post-modulation assessment, which included the resting-state EEG and the VSC Task

We employed a randomized, triple-blind, sham-controlled design. We considered within-subject factors, including time (pre-/post-intervention) and condition (neutral/reward), as well as a between-subject factor, which was the group (active/sham tDCS). We preregistered our detailed research plan based on our pilot study on Open Science Framework (10.17605/OSF.IO/5HVPW). When a participant arrived at our laboratory, they read the information letter, checked the exclusion criteria, and signed the informed consent form. Subsequently, they were seated in a comfortable chair in a dimly lit testing room for the placement of EEG electrodes on the scalp. After placing the electrodes, we collected resting EEG data, with blocks of both EO and EC sessions. Following the resting EEG recording, participants completed the questionnaires. Subsequently, they proceeded to the first part of the VSC Task before the tDCS intervention. EEG was recorded throughout the resting states and computer tasks, but not during the stimulation as the cap was changed. Subsequently, they were randomly assigned to receive either active or sham tDCS. The tDCS software provided by Neuroelectrics (www.neuroelectrics.com) completed the assignments, ensuring that the individuals involved, including the participants, experimenters, and data analysts, remained unaware of who was actually receiving the stimulation. After the tDCS intervention, we conducted the same procedure again for the post-modulation assessment, including the resting-state EEG and the VSC Task. Please consider Fig. 1B for more details. The pre-/post-assessments and the intervention all took place on the same day, lasting approximately five hours. Analyses were conducted anonymously.

### Visuospatial cueing task (VSC)

The VSC task was presented using OpenSesame [35]. It was an adapted version from the original [1] and included an intrinsic reward condition based on previous research [21, 36] in addition to the neutral condition. Figure 1A illustrates the details of the task. Trials were randomized, and the condition order (neutral/reward) was counterbalanced across the participants. Only the first keyboard response to a target was logged for each trial. There were four main blocks (two for each condition) with a short practice block at the beginning of the task, and it took approximately 45 min in total. The number of total trials is 1280 (160 trials per block, 320 trials per condition, and 640 trials per phase, i.e., either pre-intervention or post-intervention). Participants were seated approximately 65 cm from the screen. The task began with clear instructions and verbal explanations when needed. Throughout the task, participants were instructed to keep their eyes fixed on the center of the screen. Furthermore, we instructed participants to keep their heads still, and if any movement occurred, we promptly corrected their positioning. First, a fixation dot was presented for 2000 ms. Subsequently, a central cue (width: 100 (2.5°), height: 100 (2.5°)) was presented for 400 ms. The cue indicated to which side of the screen the participant had to direct their attention. In the case of neutral cues, the cues did not indicate either the right or left side of the screen. Following that, a target was presented at either the left or right visual hemifield, which could be non-cued (presented after the neutral cue), valid (congruent with the location as indicated by the cue), or invalid (incongruent with the location indicated by the cue). The target was bar-shaped and could be either short (width: 100 (2.56°), height: 120 (3.07°)) or long (width: 100 (2.56°), height: 200 (5.12°)). The required response depended on the height of the bar, and stimulus–response assignments were counterbalanced across the participants. The intertrial interval was set to 3100 ms. The neutral and reward contexts differed in terms of the targets. We used stimuli with moderate contrast in the neutral condition, grey bar-shaped targets. Previous research guided us in stimuli selection [14]. In the intrinsic reward condition, the bar-shaped targets represented palatable food images, such as chips, chocolate, cookies, and cashew nuts. These images are easy to recognize and are high in fat and sugar. They are regarded as intrinsic rewards by both healthy and clinical samples [21, 36]. Attentional bias was computed by subtracting the mean RT to valid targets from non-cued targets. A larger value indicates a stronger attentional bias. Attentional disengagement was computed by subtracting the RT to non-cued targets from invalidly cued targets. A larger value indicates lower attentional disengagement.

### EEG recording

Scalp voltages were recorded using the NeXus-32 from Mind Media [37], employing a 21-channel cap Ag/AgCl electrodes placed according to the 10–20 system. The sampling frequency was 512 Hz, and activity was referenced to linked-ears. The horizontal electrooculography (HEOG) was recorded bipolarly from the outer canthi of both eyes, and the vertical EOG (VEOG) was recorded supra-relative to infra-orbital. EEG was recorded continuously except during tDCS-application.

### Transcranial direct current stimulation (tDCS)

**Fig. 2:**
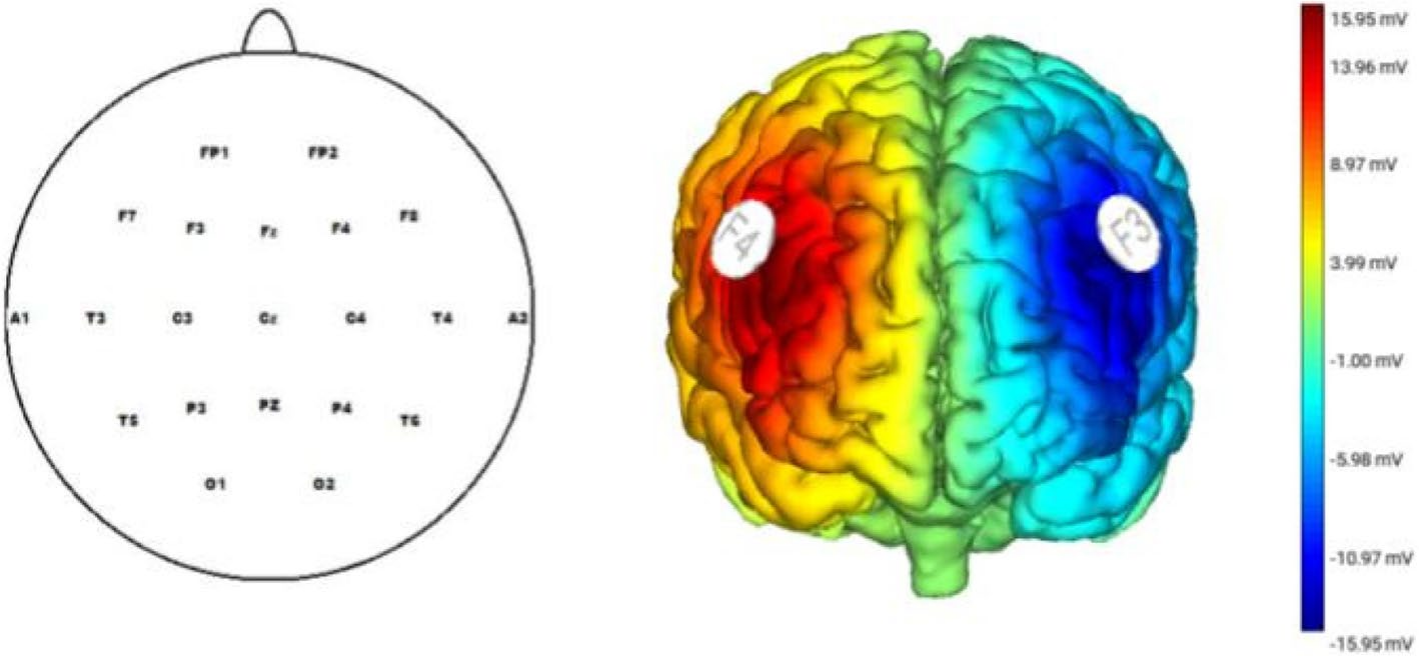
The figure illustrates the electrode sites used in our study, based on the 10–20 system. Frontal alpha asymmetry was computed by subtracting mean alpha oscillatory activitiy at F3 from F4, and F7 from F8. Additionally, we examined the early directing attention negativity (EDAN) at P4/P3 and the late directing attention positivity (LDAP) at T4/T3. Furthermore, we investigated the N1 and P1 effects at P3 and P4 (averaged), and finally, the late positive difference (LPD) at Cz. B) This figure shows the placement of the tDCS electrodes and the protocol. A constant current of 2 mA was applied via two circular sponge electrodes (25 cm^2^), placed over positions F4 (anode) and F3 (cathode) with saline solution. The maximal electric field strength was 15.95 µV for the anodal electrode

Brain modulation aimed to increase the activity of the right relative to the left DLPFC. Direct electrical current was transmitted using a pair of saline-soaked, circular sponge electrodes (25 cm^2^) and delivered via STARSTIM-8, Neuroelectrics (www.neuroelectrics.com). The anode was placed on the right DLPFC (F4), while the cathode was placed on the left DLPC (F3), based on the 10–20 system. A constant current of 2 mA was applied for 20 min [9] (Fig. 2) unless it was a sham condition. We had a 30-s ramp-up to 2 mA and 30-s ramp-down. In the sham condition, a brief current was applied with only ramp-up and ramp-down at the beginning of the stimulation session to ensure that the sensory experience was comparable to that in the active condition. The employed tDCS procedures have been demonstrated to be safe in healthy participants [38]. Following the conclusion of the experiment, participants were asked with determining the condition in which they had been placed. Approximately 43.5 percent of the participants made incorrect guesses about their conditions, with 46.8 percent of those in the active group providing inaccurate responses.

### Statistical analyses

We used BrainVision Analyzer 2 (www.brainproducts.com) to preprocess the electrophysiological data. FAA scores were calculated based on resting EEG states collected during 5 min of eyes open (EO) and 5 min of eyes closed (EC) sessions before and after the intervention. By comparing EEG resting-state recordings in these two conditions, it is easier to understand the impact of sensory input, internal cognitive processes, and the brain’s intrinsic activity, leading to a comprehensive understanding of the brain’s functional organization in different states [39]. Congruent with previous research [11], FAA was calculated as follows. Prior to performing independent component analysis (ICA), as recommended by Luck [40], we applied a high-pass filter at 0.5 Hz, a low-pass filter at 40 Hz, and a 50 Hz notch filter. To minimize potential artifacts, we excluded the first and last 10 s of each approximately 5-min segment. These segments were subsequently divided into 2-s epochs. For the EO condition, ocular artifacts were corrected using ICA based on signals from the VEOG and HEOG electrodes. The segments were baseline corrected using the −100 – 0 ms interval and whole-segment baseline corrected. Epochs containing any remaining artifacts (based on 75 µV max amplitude ± relative to baseline criterion) were discarded, and Power Spectral Density (PSD) was calculated using Fast Fourier Transform FFT with a 10% Hanning window. Afterwards, the epochs were averaged and mean activity in the alpha frequency band (8–13 Hz) was calculated, and values were exported from F3, F4, F7, and F8. Alpha power was corrected for skew via natural log transform using SPSS 22 [41]. Lastly, frontal asymmetry was calculated by subtracting the mean of log-transformed alpha at lateral left electrode sites from right electrode sites (F4-F3 and F8-F7) based on Smith et al. [11]. Thus, a higher FAA value reflects higher left frontal activity (i.e., lower left frontal alpha power).

ERP signals were referenced to linked mastoids. Then, following previous experiments [42, 43] and guidelines (e.g., Handy [44]), EEG data were filtered (offline) using a low pass filter of 30 Hz, a high pass filter of 0.1592, and a notch filter of 50 Hz. Data were segmented into epochs ranging from −100 ms to 2300 ms cue-locked and from −100 to 1000 ms target-locked. Epochs were corrected for blinks (VEOG) using the Gratton et al. [45] algorithm. Segments with evidence of horizontal eye movement higher than 60 µV were rejected [16]. Subsequently, epochs were baseline-corrected with the baseline set at −100 to 0 ms. The cue-locked ERPs indicated previously were calculated in accordance with van der Lubbe et al. [16]. The activity at ipsilateral electrodes was subtracted from the activity at contralateral electrodes relative to the cue direction for each participant. The EDAN and LDAP were quantified in the 240–280 ms and 560–640 ms time window at electrode pairs P3/P4 and T3/T4, respectively. However, after visual inspection of grand average waveforms, the latency of the 240–280 ms time window for the EDAN was adjusted to 190–250 ms. Based on Mangun and Hillyard [14], the P1 and N1 were analyzed at parietal electrodes P3 and P4 and quantified as the mean amplitude in time windows 100–137 ms and 141–188 ms, respectively, while the LPD was quantified as the mean amplitude in 229–299 ms time window at Cz electrode. The P1, N1, and LPD *effects* were computed by subtracting the mean activity within the aforementioned time windows relative to invalidly cued target onset from the activity relative to validly cued target onset.

The inferential statistical analyses were conducted using SPSS 22 [41] and R [46]. First, participants with missing values and outliers exceeding 3 *SD* from the mean were excluded in case of evident erroneous data. Then, we used repeated measures ANOVA to test the hypotheses. The electrophysiological variables under investigation included FAA (EO/EC), EDAN, LDAP, N1 *effect,* P1 *effect*, and LPD *effect*, as well as the behavioral indices of attentional bias and disengagement. For all analyses, the significance level was set at 0.05. We employed a 2 × 2 design (Time x Group) for FAA models, whereas the other models followed a 2 × 2 × 2 structure (Time x Condition x Group). We also performed a series of Bayesian repeated measures ANOVA for the null results. They were used with Bayes factor 01 (BF_01_), which is in favor of null hypotheses over alternative hypotheses. More particular, a score of less than 0.33 indicates strong support for alternative hypotheses [47, 48]. Secondary analyses were performed on the behavioral and ERP indices of attentional processes, excluding participants who showed an absent validity effect at baseline in the VSC task. Additionally, exploratory analyses were conducted to explore the effects of time and group in the reward condition. We did not perform a correction for multiple comparisons. Indeed, the inclusion of the additional secondary analyses increases the risk of false positives. However, these analyses were exploratory, and applying the correction for multiple comparisons could have reduced the sensitivity to detect potential effects in our primary outcomes. Hence, we decided to omit this correction. As a result, and inherent to exploratory analyses, the results of these analyses should be interpreted with caution. For transparency, detailed results from exploratory and Bayesian analyses are included in the supplementary materials.

## Results

### Results regarding the transcranial direct current stimulation and frontal alpha asymmetry

**Fig. 3:**
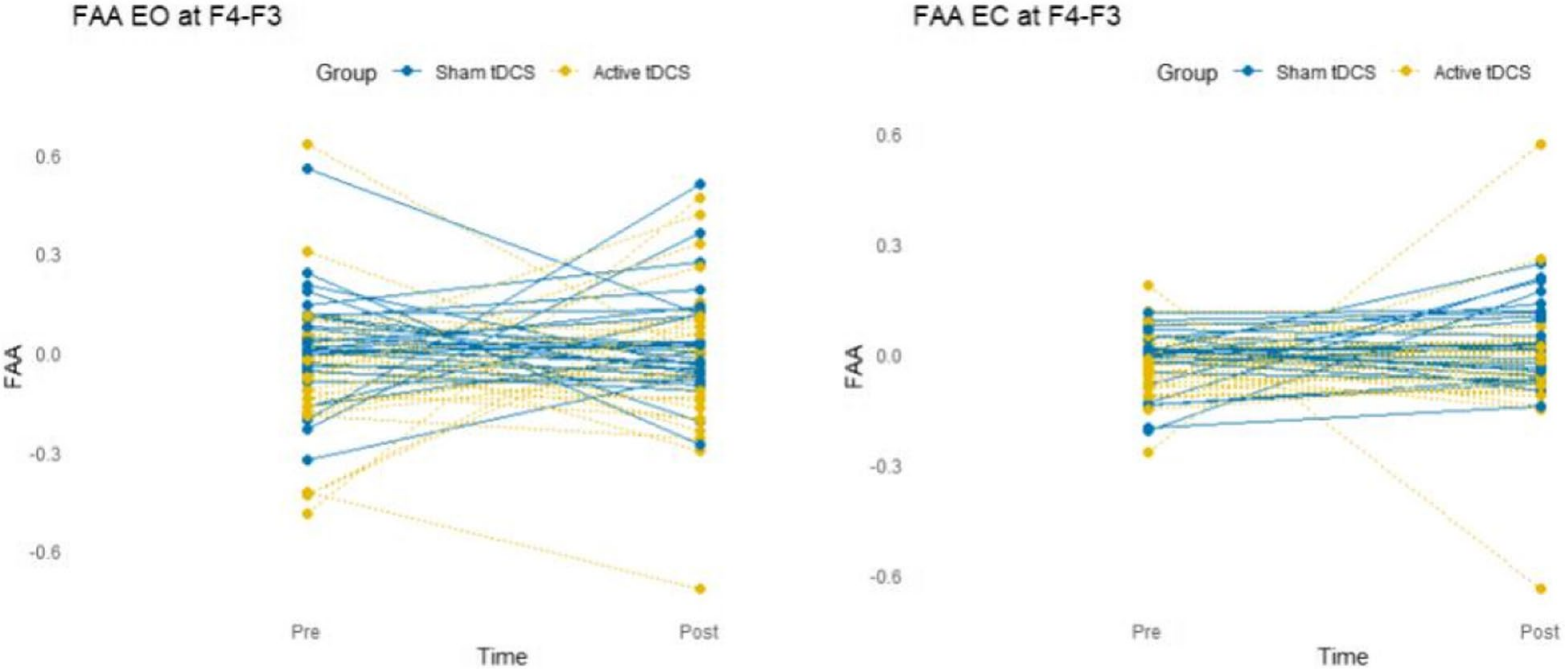
Individual pre- to post-intervention changes in FAA, both for the eyes open (EO) and eyes closed (EC) conditions

To test whether the tDCS intervention affected the primary target resting-state FAA, a series of repeated measures of ANOVA with the within-subjects factor of time (pre-/post-intervention) and the between-subject factor of group (active/sham tDCS) was conducted. The frequentist and Bayesian analyses showed that there is no effect of time and group interactions on FAA F4-F3 (EO): *F*(1, 114) = 0.28, *p* = 0.594, η^2^p = 0.002 and on FAA F4-F3 (EC): *F*(1, 114) = 0.49, *p* = 0.484, η^2^p = 0.004. Bayesian results for various models are in favor of the null hypothesis (BF01 > 0.3). These results were previously reported by another study in detail [49]. Please refer to the supplementary materials for more details. In addition to these previous findings, and in order to depict individual variation, we also plotted pre- to post-intervention changes per participant in Fig. 3.

### Results regarding the transcranial direct current stimulation and behavioral indices of visuospatial attention

**Fig. 4:**
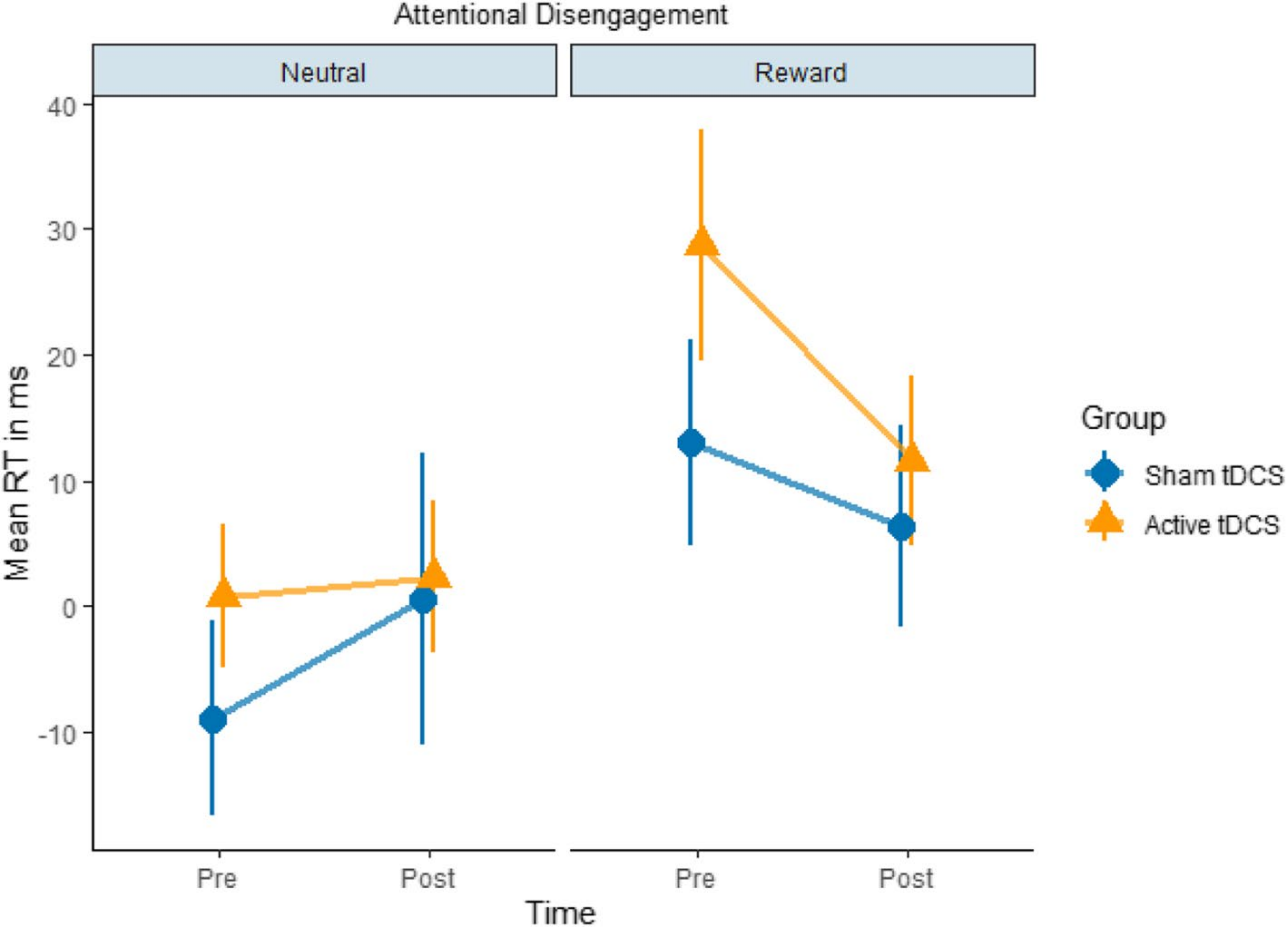
This figure shows the mean RTs of attentional disengagement based on time (pre-/post-intervention), condition (neutral/reward), and group (sham/active tDCS) factors. It depicts reduced attentional disengagement in the reward condition compared to the neutral condition. Please note that attentional disengagement was calculated by subtracting the mean RT to non-cued targets from invalidly cued targets. Therefore, a greater value indicates lower attentional disengagement

We conducted a series of repeated measures ANOVA with the within-subject factors of time (pre-/post-intervention) and condition (neutral/reward), as well as the between-subject factor of group (active/sham tDCS). The statistical results are provided in Table 1. Although there was a significant main effect of condition on attentional disengagement, *F*(1, 232) = 8.59, *p* = 0.003, η^2^p = 0.035, its interaction with time and group factors was not significant, *F*(1, 232) = 0.01, *p* = 0.909, η^2^p = < 0.001. Similarly, the Bayesian models provided strong evidence regarding the main effect of condition, BF_01_ < 0.3 (Table 7 in the supplementary materials). Please refer to Tables 6 and 7 in the supplementary materials for further information. The direction of the relationship between attentional disengagement and condition is illustrated in Fig. 4. The figure showed reduced attentional disengagement in the reward condition compared to the neutral condition.

**Table 1:** Results of attentional bias and attentional disengagement models

Variables	*df*	*F*	*p*	η^2^p
Attentional Bias (*n* = 60)				
Time	1	0.01	0.917	< 0.001
Condition	1	2.60	0.108	0.011
Group	1	2.05	0.153	0.008
Time x Condition	1	0.18	0.670	< 0.001
Time x Group	1	1.22	0.270	0.005
Condition x Group	1	0.03	0.851	< 0.001
Time x Condition x Group	1	0.11	0.730	< 0.001
Error	232			
Attentional Disengagement (*n* = 60)				
Time	1	0.42	0.517	0.001
Condition	1	8.59	0.003*	0.035
Group	1	2.05	0.153	0.008
Time x Condition	1	2.44	0.119	0.010
Time x Group	1	0.67	0.413	0.002
Condition x Group	1	0.17	0.677	< 0.001
Time x Condition x Group	1	0.01	0.909	< 0.001
Error	232			

*0.05

Participants with missing values and erroneous data were excluded from the analyses

### Results regarding the transcranial direct current stimulation and cue-locked event-related potentials

**Fig. 5:**
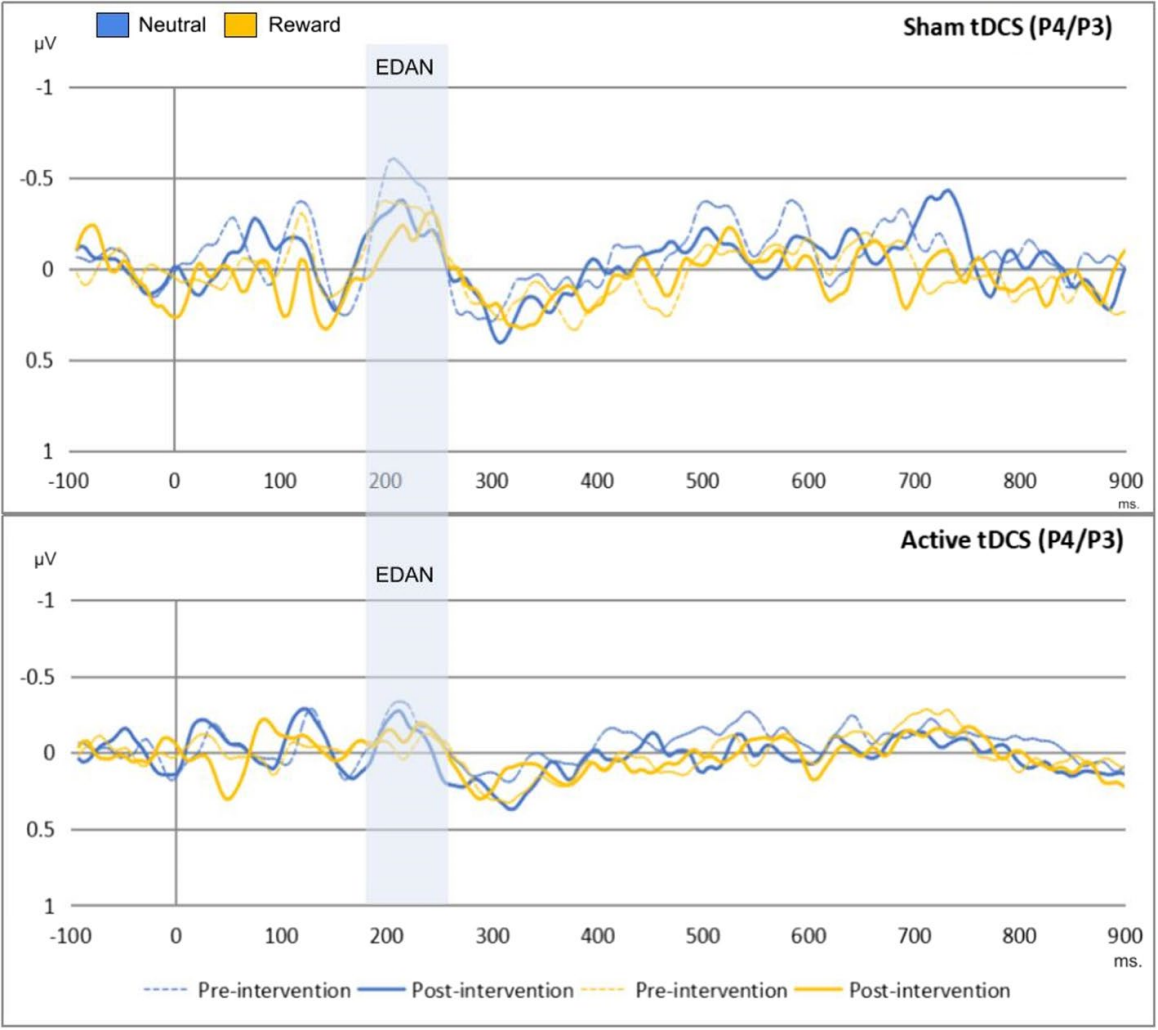
This figure shows the effects of time, condition, and group on the EDAN (190–250 ms). The x-axis represent the time in milliseconds, while the y-axis represents the EDAN scores in microvolts. Based on the depiction, the EDAN was replicated in the study regardless of time, condition, and group effects

**Fig. 6:**
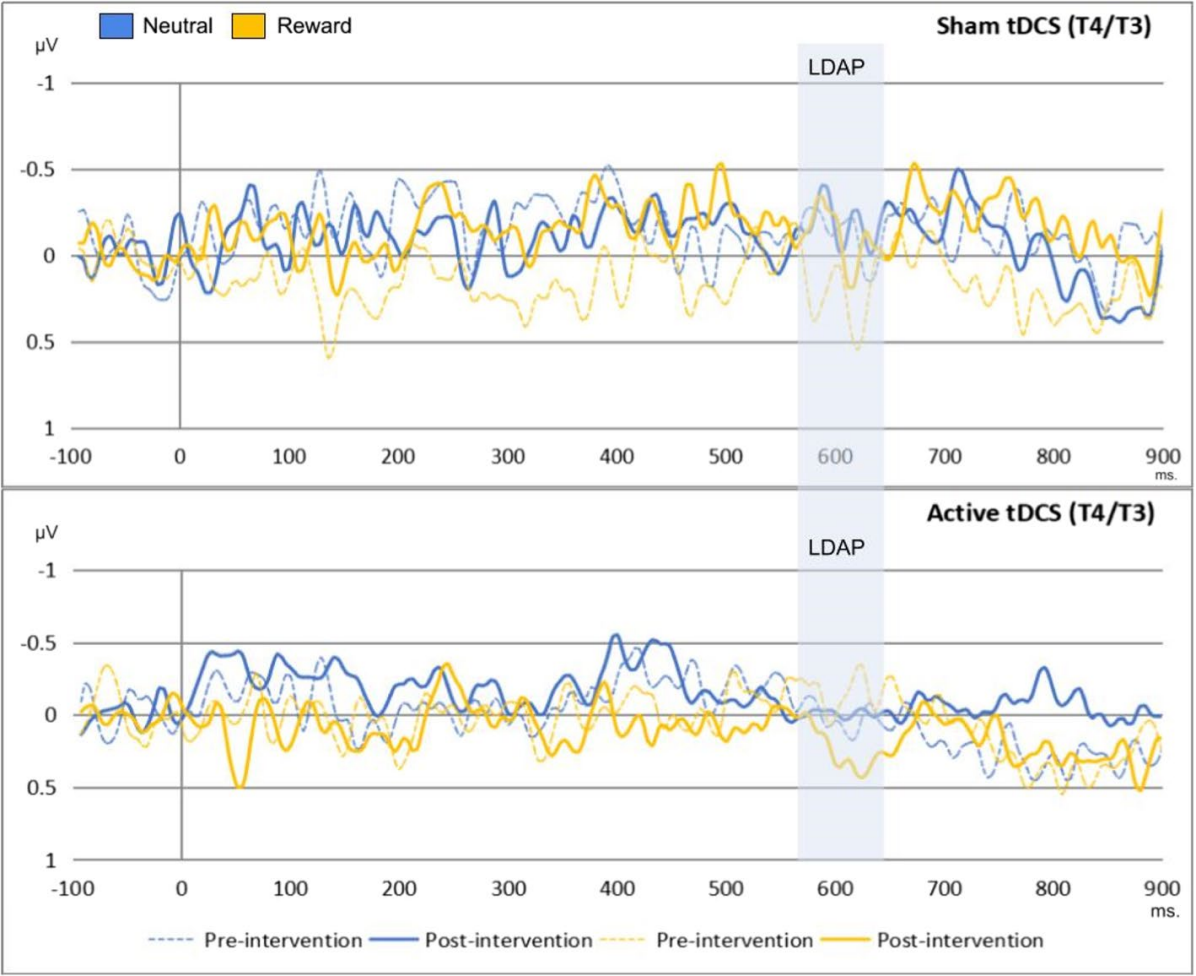
This figure shows the effects of time, condition, and group on the LDAP (560–640 ms). The x-axis represents the time in milliseconds, while the y-axis represents the LDAP scores in microvolts. Based on the depiction, the LDAP was replicated in the study

**Fig. 7:**
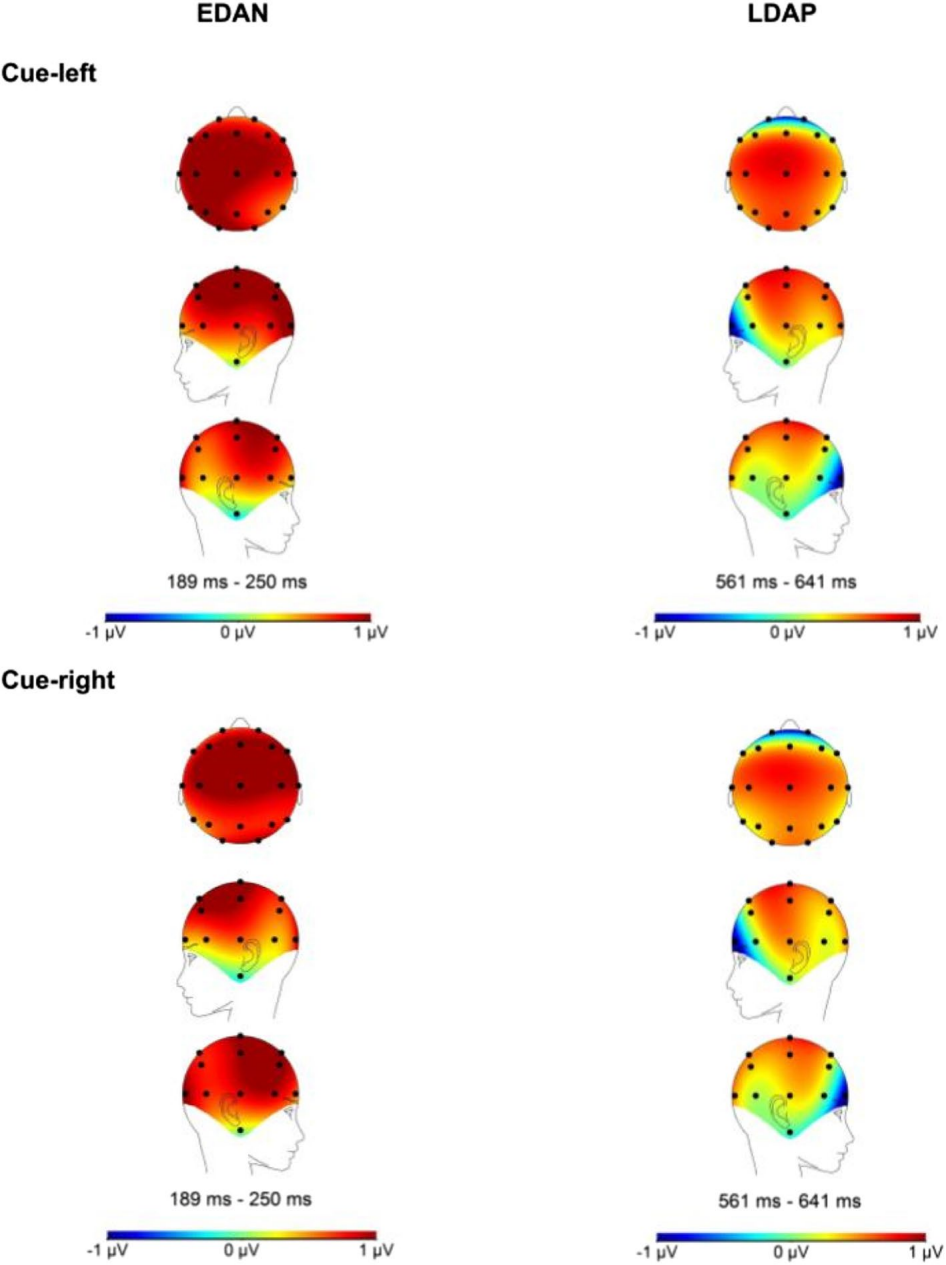
This figure illustrates the topographic activity based on baseline conditions (pre-intervention/neutral condition) for cue-locked event-related potentials

A series of repeated measures ANOVA was conducted on each cue-locked ERP. The within-subject factors included time (pre-/post-intervention) and condition (neutral/reward), while the between-subject factor was group (active/sham tDCS). The results concerning the EDAN and LDAP can be observed in Figs. 5 and 6, respectively. While Table 2 shows the rejected trials per channel, Fig. 7 displays the topographic activity during baseline conditions, specifically under pre-intervention neutral conditions. This visualization highlights the distribution of neural activity. For detailed inferential statistics, refer to Table 3. We found that the effect of time, condition, and group interactions on LDAP showed a trend towards significance, *F*(1, 56) = 2.98, *p* = 0.090, η^2^p = 0.051. Similarly, we found a trend towards significance in the effect of time and group interactions on EDAN, *F*(1, 56) = 3.09, *p* = 0.084, η^2^p = 0.052. These results were supported by Bayesian analyses with anecdotal to strong evidence for null hypotheses, and they can be found in Tables 8 and 9 in the supplementary materials.

**Table 2:** Rejection rates for each event-related potential channels

Variables	Pre		Post	
	Neutral	Reward	Neutral	Reward
	(%)	(%)	(%)	(%)
Cue-locked channels (*n* = 58)				
P3	0.81	1	0.75	0.89
P4	0.81	1.48	0.81	1.14
T3	1.74	2.57	1.36	1.57
T4	1.18	1.79	1.35	1.13
Target-locked channels (*n* = 55)				
P3	0.79	1.25	0.58	0.64
P4	0.76	1.22	0.59	1.1
Cz	0.74	1.29	1.14	0.8

“Pre” indicates pre-intervention, while “post” represents post-intervention

**Table 3:** Results of cue-locked event-related potential models

Variables	*df*	*F*	*p*	η^2^p
EDAN at 190–250 ms. (P4/P3) (*n* = 58)				
Time	1	2.13	0.149	0.037
Condition	1	0.71	0.402	0.013
Group	1	0.71	0.401	0.013
Time x Condition	1	0.69	0.408	0.012
Time x Group	1	3.09	0.084	0.052
Condition x Group	1	< 0.01	0.991	< 0.001
Time x Condition x Group	1	0.28	0.597	0.005
Error	56			
LDAP at 560–640 ms. (T4/T3) (*n* = 58)				
Time	1	0.11	0.733	0.002
Condition	1	0.01	0.896	< 0.001
Group	1	0.02	0.874	< 0.001
Time x Condition	1	< 0.01	0.958	< 0.001
Time x Group	1	0.01	0.899	< 0.001
Condition x Group	1	< 0.01	0.923	< 0.001
Time x Condition x Group	1	2.98	0.09	0.051
Error	56			

Participants with missing values and erroneous data were excluded from the analyses

### Results regarding the transcranial direct current stimulation and target-locked event-related potentials

**Fig. 8:**
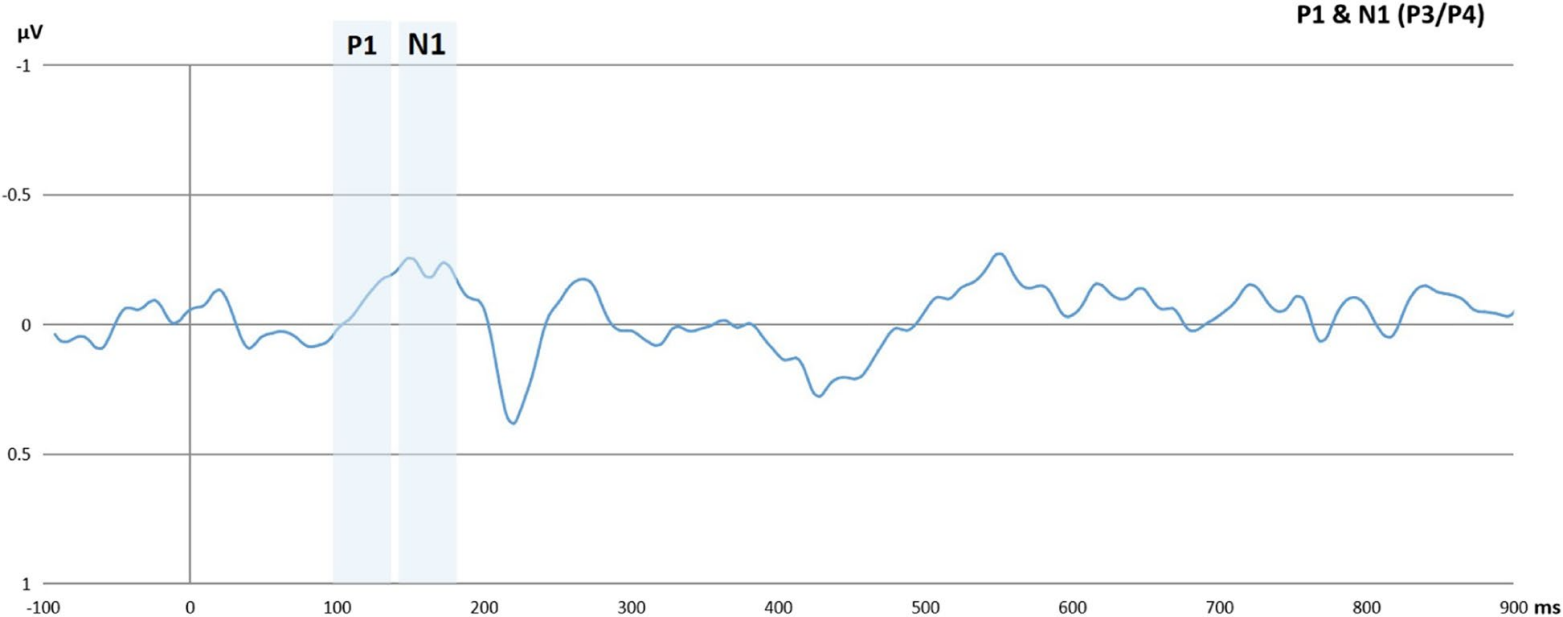
The figure shows the overall P1 *effect* and N1 *effect* collapsed over time, group, and condition*.* The x-axis represents the time in milliseconds, while the y-axis represents the P1 *effect* and N1 *effect* scores in microvolts. The P1 effect was calculated by subtracting the P1 to invalid trials from the P1 to valid trials. Similarly, the N1 was calculated by subtracting the N1 to invalid trials from the N1 to valid trials

**Fig. 9:**
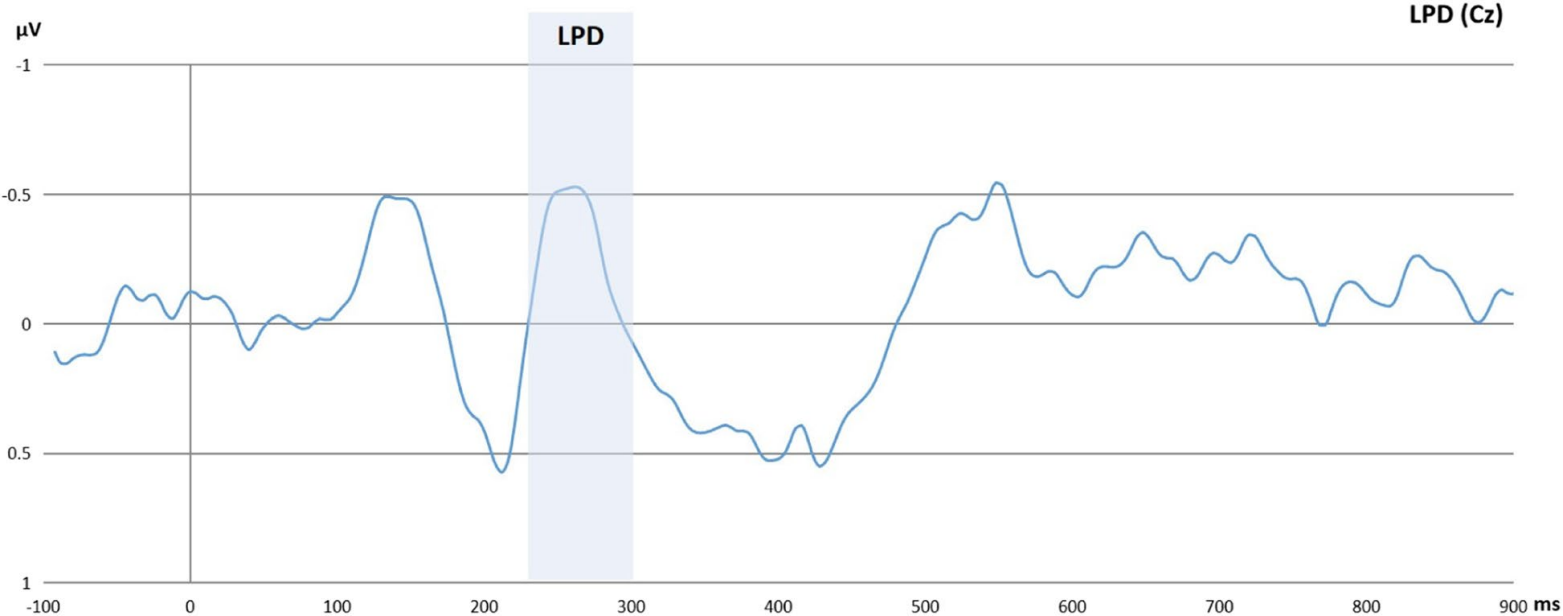
The figure shows the overall LPD *effect* collapsed over time, group, and condition. The x-axis represents the time in milliseconds, while the y-axis represents the LPD *effect* scores in microvolts. The LPD *effect* was calculated by subtracting the LPD to invalid trials from the LPD to valid trials. Therefore, the negative peak represents an enhanced LPD for invalid trials relative to valid trials as well as a reduced LPD for valid trials relative to invalid trials

**Fig. 10:**
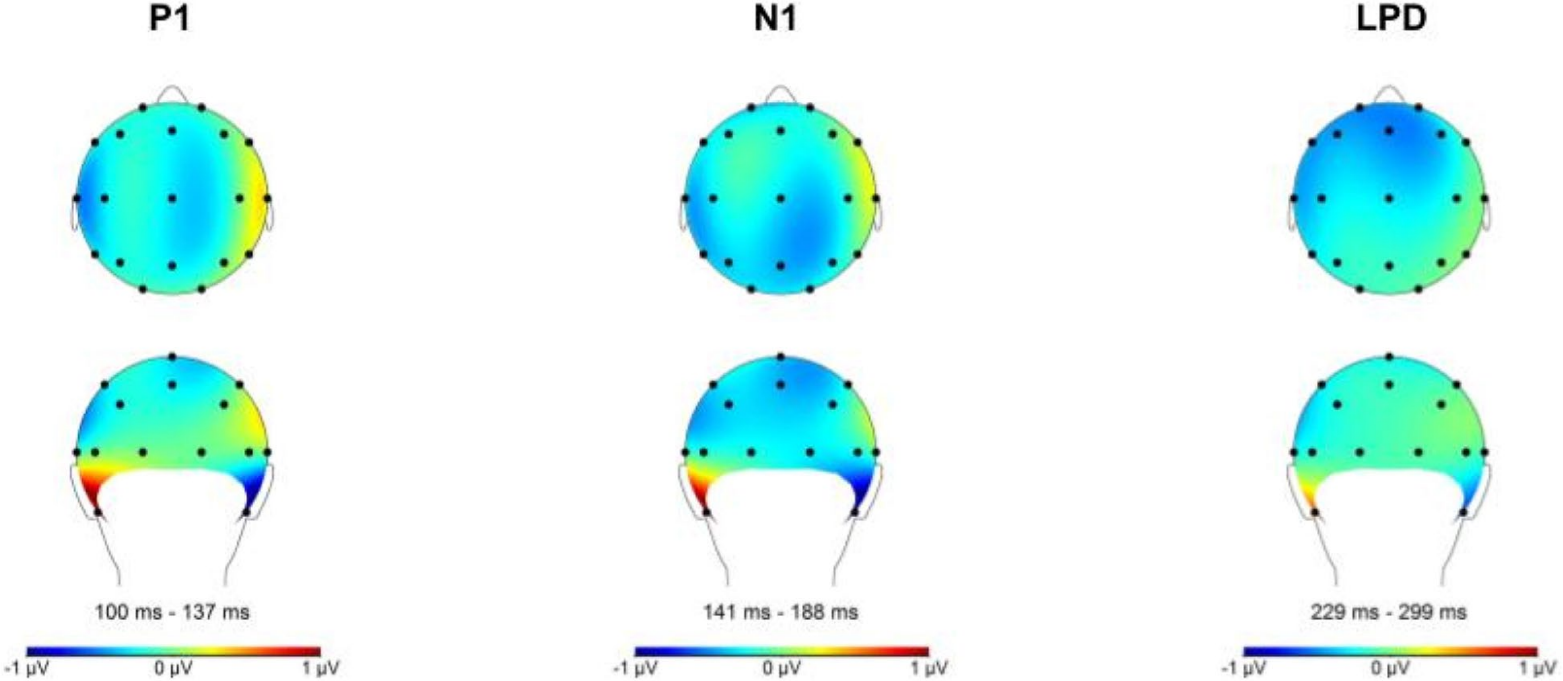
The figure shows the overall topographic activity for target-locked event-related potentials, with time, condition, and group collapsed

We conducted a series of repeated measures ANOVA to assess whether the tDCS intervention influenced the target-locked ERPs. The within-subject factors for the models were time (pre-/post-intervention) and condition (neutral/reward), while the between-subject factor was group (active/sham tDCS). We found no significant effect of time, condition, and group interactions on target-locked ERPs as shown in Tables 4, 5, and 6 for the P1 *effect*, N1 *effect*, and LPD *effect*, respectively. These results are also illustrated in Figs. 8 and 9. Moreover, Fig. 10 shows the topographic activity. Similar to previous explorations, the P1 *effect* was not identified [43]. Overall, as shown in Tables 5 and 6, the N1 and LPD modulations were significant, and these components were clearly discernible in the figures. The Bayesian models provided anecdotal to strong evidence in favor of the null hypotheses: Please refer to Tables 10, 11, 12, 13, 14 in the supplementary materials for Bayesian results.

**Table 4:** Results of P1 effect models

Variables	*df*	*F*	*p*	η^2^p
P1 at 100–137 ms. (P3) (*n* = 55)				
Intercept		1.25	0.269	0.023
Time	1	1.85	0.179	0.034
Condition	1	0.05	0.818	0.001
Group	1	0.90	0.347	0.017
Time x Condition	1	1.09	0.084	0.055
Time x Group	1	0.19	0.662	0.004
Condition x Group	1	0.82	0.368	0.015
Time x Condition x Group	1	0.37	0.545	0.007
Error	53			
P1 at 100–137 ms. (P4) (*n* = 55)				
Intercept		0.79	0.375	0.015
Time	1	2.60	0.113	0.047
Condition	1	0.25	0.257	0.024
Group	1	1.37	0.246	0.025
Time x Condition	1	2.40	0.127	0.043
Time x Group	1	1.31	0.257	0.024
Condition x Group	1	3.88	0.054	0.068
Time x Condition x Group	1	0.01	0.896	< 0.001
Error	53			

Participants with missing values and erroneous data were excluded from the analyses

**Table 5:** Results of N1 effect models

Variables	*df*	*F*	*p*	η^2^p
N1 at 141–188 ms. (P3) (*n* = 55)				
Intercept	1	2.96	0.091	0.053
Time	1	2.25	0.140	0.041
Condition	1	0.18	0.666	0.004
Group	1	1.20	0.277	0.022
Time x Condition	1	1.28	0.262	0.024
Time x Group	1	< 0.01	0.994	< 0.001
Condition x Group	1	< 0.01	0.936	< 0.001
Time x Condition x Group	1	0.78	0.379	0.015
Error	53			
N1 at 141–188 ms. (P4) (*n* = 55)				
Intercept	1	8.59	0.005*	0.140
Time	1	1.42	0.238	0.026
Condition	1	< 0.01	0.940	< 0.001
Group	1	0.44	0.509	0.008
Time x Condition	1	1.51	0.224	0.028
Time x Group	1	0.99	0.324	0.018
Condition x Group	1	0.15	0.695	0.003
Time x Condition x Group	1	0.15	0.694	0.003
Error	53			

*0.05

Participants with missing values and erroneous data were excluded from the analyses

**Table 6:** Results of the LPD effect model

Variables	*df*	*F*	*p*	η^2^p
LPD at 229–299 ms. (Cz) (*n* = 55)				
Intercept	1	6.62	0.013*	0.111
Time	1	3.37	0.072	0.060
Condition	1	0.34	0.559	0.006
Group	1	1.26	0.267	0.023
Time x Condition	1	2.86	0**.**097	0.051
Time x Group	1	0.11	0.734	0.002
Condition x Group	1	1.66	0.202	0.030
Time x Condition x Group	1	0.14	0.703	0.003
Error	53			

*0.05

Participants with missing values and erroneous data were excluded from the analyses

### Exploratory analyses

In line with a previously reported approach [50], secondary analyses were conducted with individuals who showed a validity effect at baseline in both neutral and reward conditions. As stated previously, the validity effect was calculated based on higher RTs in invalid trials than in valid trials (invalid trials > valid trials). The following analyses were conducted based on this selected sample.

Similar to the previous results, the main effect of condition on attentional disengagement was significant, *F*(1, 128) = 4.86, *p* = 0.029, η^2^p = 0.034. Indeed, secondary analyses based on the validity effect did not yield any additional information regarding the behavioral indices of visuospatial attention. Please see Table 15 in the supplementary file for further information.

**Fig. 11:**
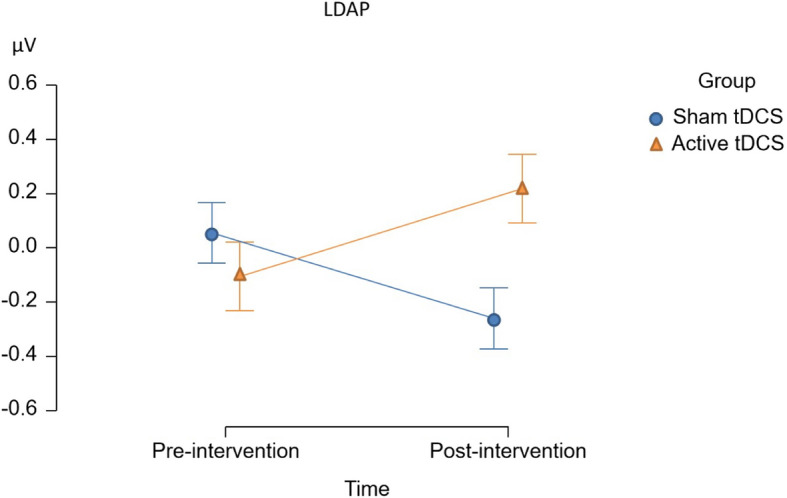
The figure shows the exact effect of time and group interactions on the LDAP in the reward context. The x-axis represents the time factor, while the y-axis represents the LDAP in microvolts

**Fig. 12:**
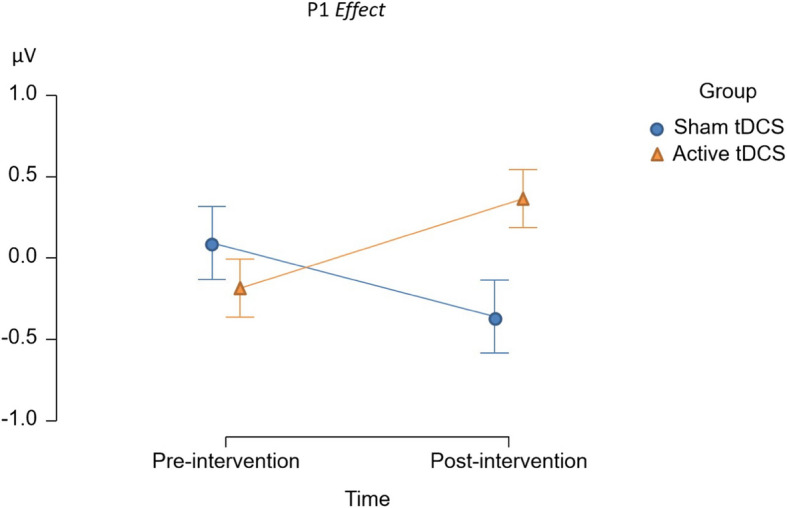
The figure shows the exact effect of time and group interactions on the P1 effect in the reward context. The x-axis represents the time factor, while the y-axis represents the P1 effect in microvolts

We also tested the effect of time and group interactions on electrophysiological indices of visuospatial attention in the neutral and reward conditions. Both the LDAP and P1 *effect* were enhanced in the active tDCS group relative to the sham tDCS group in the reward context, *F*(1, 30) = 6.70, *p* = 0.015, η^2^p = 0.183 and *F*(1, 30) = 6.30, *p* = 0.018, η^2^p = 0.174, respectively. They can also be seen in Figs. 11 and 12, respectively. Please also consider Tables between 16 and 24 in the supplementary file for more information.

## Discussion

The present study investigated the relationship between FAA as a potential neurophysiological index of asymmetric frontal cortical activity, and behavioral and brain activity indices of visuospatial attention under the influence of intrinsic reward-related stimuli, specifically palatable food images. The results revealed no significant effect of tDCS on FAA. On the other hand, the exploratory analyses regarding the electrophysiological indices of visuospatial attention demonstrated a statistically significant enhancement of attentional orienting towards food stimuli in the active tDCS group compared to the sham tDCS group. Nevertheless, these effects did not translate into observable behavioral changes. We only observed a statistically significant main effect of condition on the reaction times, indicating reduced attentional disengagement in the reward condition compared to the neutral condition.

Previous studies suggested that tDCS may affect the asymmetry of frontal brain activity [28]. Despite their relevance, these measures are considered indirect. Importantly, to the best of our knowledge, prior studies have not thoroughly investigated whether tDCS affects FAA. The results did not show any support for tDCS-induced shifts in the asymmetry of frontal brain activity. Though contrary to the hypothesis, the lack of a significant effect may not be too surprising. The path of electric currents is complex, involving the electrodes, scalp, skull, meninges, and cerebrospinal fluid before reaching the brain. However, each brain has a unique anatomical structure, resulting in different magnitudes and directions of the electric field in each subject [51–53]. Indeed, not only neuronal structure, such as skull thickness, sulcal depth, and gyral depth, can influence the electric field strength, but also there may be “hotspots” that resist electrode positioning [53]. Especially when compared to motor cortical tDCS, frontal tDCS produces more variable electric fields [54]. This variability may lead to conflicting results in brain stimulation studies [55]. Aside from the variations in individual brain anatomy, the effects of stimulation can also be influenced by factors such as electrode properties, including size and shape, and conductivities, such as gels and saline solutions [56]. Therefore, these findings in the literature suggest that the lack of a significant effect of tDCS on FAA may be attributed to the variability in individual brain structures, the influence of electrode positioning, and the diverse effects of tDCS montages and electrode properties on stimulation outcomes. Future studies should take into account and control for individual differences to ensure the validity and reliability of the findings.

Even though primary analyses did not reveal a significant effect of tDCS on attentional bias and attentional disengagement, via FAA, at the behavioral level, the results indicated a potential effect of tDCS on the electrophysiological measure of attentional orienting, which was confirmed in our exploratory analyses. For these analyses, we employed the same approach as in Thiel et al. [50] and excluded participants who did not show evidence of cue-induced manipulation of attention. In the resulting sample, tDCS resulted in an enhanced cue-induced LDAP, specifically in the reward context. This can be interpreted as a tDCS-induced enhancement of top-down attentional orienting to spatial locations associated with upcoming reward-associated stimuli. This effect contradicts the hypothesized shift toward enhanced right versus left frontal activity in our study and previous studies. More specifically, previous research has reported that tDCS enhances inhibitory control [28]. The effect of tDCS on enhancing inhibitory control might arguably outweigh its impact on attentional bias and any potential associated response complicating inhibitory control. Therefore, the question arises as to which alternative mechanism fits the observed effect. Likewise, a study found positive effects on inhibitory control irrespective of the stimulation site, pointing towards a more general mechanism: Both anode right/cathode left and anode left/cathode right tDCS increased inhibitory control [28]. The answer to these questions can potentially be found in animal studies. Animal studies suggest that tDCS generates a sub-threshold depolarization of neuronal membrane potential, leading to an increased likelihood of neuronal discharge and/or synaptic transmission [57–59]. It was reported that tDCS induces cerebral plasticity based on calcium (Ca2 +) elevation of astrocytic origin in mice. This tDCS-induced Ca2 + elevation is blocked by noradrenergic neuron ablation. Together, these imply that tDCS results in increased Noradrenaline (NA) release [57–59]. The question is whether enhanced attentional orienting and inhibitory control can be explained by increased noradrenergic neurotransmission. Previous studies in psychopharmacology lend support to this notion. In a study, noradrenergic challenge with Clonidine resulted in a reduction of attentional bias, as indexed by electrophysiology. Hence, inversely, the facilitation of NA plausibly results in enhanced attentional bias, which aligns with the current observed results in the study [43]. On the other hand, another study on inhibitory control indicated that inhibitory activity (evidenced by the Stop P3) is reduced following attenuated NA neurotransmission [60]. Therefore, tDCS enhancement of NA would likely result in improved inhibitory control simultaneously.

On the other hand, the outcome of these exploratory analyses should be taken into consideration with caution due to the absence of correction for multiple comparisons and the application of a such correction for multiple comparisons would not change the interpretation of the primary results.

Lastly, the only significant behavioral effect in the experiment was the main effect of condition on attentional disengagement. We unsurprisingly found that attentional disengagement was reduced in the reward condition compared to the neutral condition regardless of group. In the absence of a significant effect from tDCS, we can expect food stimuli to pose a challenge to attentional disengagement potentially. This finding is in line with similar studies reporting that food stimuli challenge inhibitory control [36, 61].

Despite the valuable insights gained from this study, several limitations need to be addressed. First, as indicated previously, individual variations in brain anatomy, coupled with a relatively modest sample size, might have contributed to the null results, and discrepancy between frequentist and Bayesian approaches in attentional disengagement outcomes. Besides, the effectiveness of tDCS is contingent on various factors, including stimulation parameters (e.g., duration, intensity, electrode placement). While the chosen intervention parameters were based on existing literature and rationale, other stimulation settings could have yielded different results. In the current study, the tDCS intervention was administered in a single session. Multiple sessions might produce different effects on frontal brain activity, potentially influencing the observed outcomes. Although FAA was investigated as a primary mediator, this study highlights that other neurobiological/neurochemical mechanisms, such as the noradrenergic system, may underlie the observed effects regarding attentional reorienting. However, the specific mechanisms were not directly explored in this study, leaving room for further investigation. Furthermore, it is important to acknowledge that frontal asymmetry patterns may reflect activity within a broader fronto-parietal network involved in attentional control, rather than being solely attributed to DLPFC activity [62, 63]. Lastly, we did not control for differences in stimulus complexity, so we cannot entirely rule out its contribution to the observed results. However, any potential effects of complexity likely averaged out to some extent as we employed multiple stimuli per condition. Overall, while the study contributes necessary groundwork in this area, the identified limitations should be taken into consideration while interpreting the findings and for future research.

## Conclusion

In conclusion, this preregistered study explored the relationship between frontal alpha asymmetry (FAA) and attentional control by using transcranial direct current stimulation (tDCS). While tDCS did not show significant effects on FAA or behavioral attentional processes, intriguing secondary findings suggest tDCS might enhance cue-induced approach tendencies in a reward context. However, these effects did not lead to observable behavioral changes. Our study highlights the potential involvement of the noradrenergic system in the effect of tDCS on attentional orienting to reward-associated stimuli.

## Supplementary Information

Supplementary Material 1.

## Data Availability

The raw data are available at: https://cloud.adaptingminds.nl/d/s/wC2r5yTyjZb9f2QwbJIAiubqCkdwKsm8/3gu7TZ2BkMO75OwHmIxlb8h-MFJQSO-i-VbOAUtTD7go.

## Abbreviations

µV: Microvolt
ANOVA: Analysis of Variance
BF: Bayes Factor
Ca2 +: Calcium
DLPFC: Dorsolateral Prefrontal Cortex
EC: Eyes-Closed
EDAN: Early Directing Attention Negativity
EEG: Electroencephalogram
ELTE: Eötvös Loránd University
EO: Eyes-Open
EOG: Electrooculography
ERP: Even-Related Potentials
FAA: Frontal Alpha Asymmetry
FFT: Fast Fourier Transform
HEOG: Horizontal Electrooculography
Hz: Hertz
ICA: Independent Component Analysis
LDAP: Late Directing Attention Positivity
LPD: Late Positive Deflection
M: Mean
mA: Milliampere
NA: Noradrenaline
PSD: Power Spectral Density
rIFG: Right Inferior Frontal Gyrus
RT: Response Time
SD: Standard Deviation
SPSS: Statistical Package for the Social Sciences
SST: Stop Signal Task
tDCS: Transcranial Direct Current Stimulation
VEOG: Vertical Electrooculography
VSC: Visuospatial Cueing Task
