# Supplementary material for: The effect of transcranial direct current stimulation on frontal alpha asymmetry and visuospatial attention in food-reward contexts: a triple-blind randomized sham-controlled study
Source: BMC Psychol. 2025 Jul 1;13:695. doi: 10.1186/s40359-025-02972-x (PMC12220490; doi:10.1186/s40359-025-02972-x)
Supplement: Supplementary file 1 — Supplementary Material 1. [file 40359_2025_2972_MOESM1_ESM.docx]

| **Table 6. Results of Bayesian repeated measures ANOVA for attentional bias** | | | | | | | | | | | |
| --- | --- | --- | --- | --- | --- | --- | --- | --- | --- | --- | --- |
| **Models** | | **P(M)** | | **P(M\|data)** | | **BF _M_** | | **BF _01_** | | **error %** | |
| Null model (incl. subject) |  | 0.053 |  | 0.339 |  | 9.216 |  | 1.000 |  |  |  |
| Condition |  | 0.053 |  | 0.275 |  | 6.829 |  | 1.231 |  | 3.471 |  |
| Group |  | 0.053 |  | 0.117 |  | 2.386 |  | 2.893 |  | 2.465 |  |
| Condition + Group |  | 0.053 |  | 0.097 |  | 1.937 |  | 3.486 |  | 2.004 |  |
| Time |  | 0.053 |  | 0.047 |  | 0.891 |  | 7.179 |  | 1.140 |  |
| Time + Condition |  | 0.053 |  | 0.041 |  | 0.776 |  | 8.192 |  | 12.334 |  |
| Condition + Group + Condition  x  Group |  | 0.053 |  | 0.018 |  | 0.337 |  | 18.414 |  | 1.826 |  |
| Time + Condition + Group |  | 0.053 |  | 0.017 |  | 0.318 |  | 19.506 |  | 23.896 |  |
| Time + Group |  | 0.053 |  | 0.016 |  | 0.297 |  | 20.829 |  | 1.276 |  |
| Time + Condition + Time  x  Condition |  | 0.053 |  | 0.008 |  | 0.150 |  | 40.887 |  | 3.937 |  |
| Time + Condition + Group + Time  x  Group |  | 0.053 |  | 0.008 |  | 0.139 |  | 44.259 |  | 24.653 |  |
| Time + Group + Time  x  Group |  | 0.053 |  | 0.007 |  | 0.127 |  | 48.317 |  | 2.156 |  |
| Time + Condition + Group + Time  x  Condition |  | 0.053 |  | 0.003 |  | 0.050 |  | 122.995 |  | 2.517 |  |
| Time + Condition + Group + Condition  x  Group |  | 0.053 |  | 0.003 |  | 0.048 |  | 127.461 |  | 3.705 |  |
| Time + Condition + Group + Time  x  Condition + Time  x  Group |  | 0.053 |  | 0.001 |  | 0.023 |  | 264.941 |  | 5.880 |  |
| Time + Condition + Group + Time  x  Group + Condition  x  Group |  | 0.053 |  | 0.001 |  | 0.020 |  | 309.922 |  | 2.770 |  |
| Time + Condition + Group + Time  x  Condition + Condition  x  Group |  | 0.053 |  | 5.889e -4 |  | 0.011 |  | 575.043 |  | 4.555 |  |
| Time + Condition + Group + Time  x  Condition + Time  x  Group + Condition  x  Group |  | 0.053 |  | 2.412e -4 |  | 0.004 |  | 1404.183 |  | 4.748 |  |
| Time + Condition + Group + Time  x  Condition + Time  x  Group + Condition  x  Group + Time  x  Condition  x  Group |  | 0.053 |  | 6.981e -5 |  | 0.001 |  | 4850.799 |  | 7.689 |  |
|  | | | | | | | | | | | |
| Note:  All models include subject | | | | | | | | | | | |

| **Table 7. Results of Bayesian repeated measures ANOVA for attentional disengagement** | | | | | | | | | | | |
| --- | --- | --- | --- | --- | --- | --- | --- | --- | --- | --- | --- |
| **Models** | | **P(M)** | | **P(M\|data)** | | **BF _M_** | | **BF _01_** | | **error %** | |
| Null model (incl. subject) |  | 0.053 |  | 0.039 |  | 0.724 |  | 1.000 |  |  |  |
| Condition |  | 0.053 |  | 0.500 |  | 17.975 |  | 0.077 |  | 1.266 |  |
| Condition + Group |  | 0.053 |  | 0.175 |  | 3.807 |  | 0.221 |  | 3.290 |  |
| Time + Condition |  | 0.053 |  | 0.091 |  | 1.800 |  | 0.425 |  | 2.390 |  |
| Time + Condition + Time  x  Condition |  | 0.053 |  | 0.060 |  | 1.159 |  | 0.639 |  | 6.676 |  |
| Condition + Group + Condition  x  Group |  | 0.053 |  | 0.038 |  | 0.709 |  | 1.019 |  | 5.141 |  |
| Time + Condition + Group |  | 0.053 |  | 0.029 |  | 0.539 |  | 1.330 |  | 1.677 |  |
| Time + Condition + Group + Time  x  Condition |  | 0.053 |  | 0.019 |  | 0.341 |  | 2.077 |  | 2.566 |  |
| Group |  | 0.053 |  | 0.013 |  | 0.230 |  | 3.058 |  | 1.008 |  |
| Time + Condition + Group + Time  x  Group |  | 0.053 |  | 0.008 |  | 0.149 |  | 4.696 |  | 3.528 |  |
| Time |  | 0.053 |  | 0.007 |  | 0.122 |  | 5.724 |  | 2.478 |  |
| Time + Condition + Group + Condition  x  Group |  | 0.053 |  | 0.006 |  | 0.111 |  | 6.323 |  | 3.049 |  |
| Time + Condition + Group + Time  x  Condition + Time  x  Group |  | 0.053 |  | 0.006 |  | 0.108 |  | 6.505 |  | 7.311 |  |
| Time + Condition + Group + Time  x  Condition + Condition  x  Group |  | 0.053 |  | 0.004 |  | 0.074 |  | 9.376 |  | 4.036 |  |
| Time + Group |  | 0.053 |  | 0.002 |  | 0.039 |  | 17.764 |  | 1.503 |  |
| Time + Condition + Group + Time  x  Group + Condition  x  Group |  | 0.053 |  | 0.002 |  | 0.031 |  | 22.493 |  | 4.329 |  |
| Time + Condition + Group + Time  x  Condition + Time  x  Group + Condition  x  Group |  | 0.053 |  | 0.001 |  | 0.024 |  | 29.135 |  | 10.284 |  |
| Time + Group + Time  x  Group |  | 0.053 |  | 5.962e -4 |  | 0.011 |  | 64.817 |  | 2.395 |  |
| Time + Condition + Group + Time  x  Condition + Time  x  Group + Condition  x  Group + Time  x  Condition  x  Group |  | 0.053 |  | 5.631e -4 |  | 0.010 |  | 68.631 |  | 48.322 |  |
|  | | | | | | | | | | | |
| Note:  All models include subject | | | | | | | | | | | |

| **Table 8. Results of Bayesian repeated measures ANOVA for the EDAN** | | | | | | | | | | | |
| --- | --- | --- | --- | --- | --- | --- | --- | --- | --- | --- | --- |
| **Models** | | **P(M)** | | **P(M\|data)** | | **BF _M_** | | **BF _01_** | | **error %** | |
| Null model (incl. subject) |  | 0.053 |  | 0.398 |  | 11.904 |  | 1.000 |  |  |  |
| Time |  | 0.053 |  | 0.177 |  | 3.870 |  | 2.250 |  | 2.416 |  |
| Group |  | 0.053 |  | 0.127 |  | 2.607 |  | 3.146 |  | 2.797 |  |
| Condition |  | 0.053 |  | 0.083 |  | 1.636 |  | 4.778 |  | 4.491 |  |
| Time + Group + Time  x  Group |  | 0.053 |  | 0.059 |  | 1.122 |  | 6.783 |  | 3.145 |  |
| Time + Group |  | 0.053 |  | 0.052 |  | 0.989 |  | 7.647 |  | 1.240 |  |
| Time + Condition |  | 0.053 |  | 0.034 |  | 0.631 |  | 11.758 |  | 1.799 |  |
| Condition + Group |  | 0.053 |  | 0.023 |  | 0.431 |  | 17.019 |  | 1.306 |  |
| Time + Condition + Group + Time  x  Group |  | 0.053 |  | 0.012 |  | 0.220 |  | 33.037 |  | 5.099 |  |
| Time + Condition + Group |  | 0.053 |  | 0.010 |  | 0.190 |  | 38.089 |  | 3.604 |  |
| Time + Condition + Time  x  Condition |  | 0.053 |  | 0.008 |  | 0.153 |  | 47.203 |  | 2.610 |  |
| Condition + Group + Condition  x  Group |  | 0.053 |  | 0.005 |  | 0.085 |  | 84.496 |  | 4.513 |  |
| Time + Condition + Group + Time  x  Condition + Time  x  Group |  | 0.053 |  | 0.003 |  | 0.054 |  | 132.778 |  | 4.798 |  |
| Time + Condition + Group + Time  x  Condition |  | 0.053 |  | 0.003 |  | 0.047 |  | 152.327 |  | 3.136 |  |
| Time + Condition + Group + Condition  x  Group |  | 0.053 |  | 0.003 |  | 0.046 |  | 156.298 |  | 13.049 |  |
| Time + Condition + Group + Time  x  Group + Condition  x  Group |  | 0.053 |  | 0.002 |  | 0.039 |  | 186.447 |  | 2.162 |  |
| Time + Condition + Group + Time  x  Condition + Time  x  Group + Condition  x  Group |  | 0.053 |  | 5.614e -4 |  | 0.010 |  | 709.031 |  | 3.539 |  |
| Time + Condition + Group + Time  x  Condition + Condition  x  Group |  | 0.053 |  | 5.014e -4 |  | 0.009 |  | 793.933 |  | 2.967 |  |
| Time + Condition + Group + Time  x  Condition + Time  x  Group + Condition  x  Group + Time  x  Condition  x  Group |  | 0.053 |  | 1.459e -4 |  | 0.003 |  | 2728.194 |  | 4.265 |  |
|  | | | | | | | | | | | |
| Note:  All models include subject | | | | | | | | | | | |

| **Table 9. Results of Bayesian repeated measures ANOVA for the LDAP** | | | | | | | | | | | |
| --- | --- | --- | --- | --- | --- | --- | --- | --- | --- | --- | --- |
| **Models** | | **P(M)** | | **P(M\|data)** | | **BF _M_** | | **BF _01_** | | **error %** | |
| Null model (incl. subject) |  | 0.053 |  | 0.611 |  | 28.236 |  | 1.000 |  |  |  |
| Time |  | 0.053 |  | 0.122 |  | 2.496 |  | 5.014 |  | 25.732 |  |
| Group |  | 0.053 |  | 0.119 |  | 2.429 |  | 5.135 |  | 2.211 |  |
| Condition |  | 0.053 |  | 0.087 |  | 1.717 |  | 7.012 |  | 1.337 |  |
| Time + Group |  | 0.053 |  | 0.018 |  | 0.322 |  | 34.762 |  | 1.655 |  |
| Condition + Group |  | 0.053 |  | 0.016 |  | 0.299 |  | 37.387 |  | 1.193 |  |
| Time + Condition |  | 0.053 |  | 0.013 |  | 0.232 |  | 48.073 |  | 1.539 |  |
| Condition + Group + Condition  x  Group |  | 0.053 |  | 0.004 |  | 0.067 |  | 165.232 |  | 10.735 |  |
| Time + Group + Time  x  Group |  | 0.053 |  | 0.004 |  | 0.063 |  | 174.186 |  | 2.085 |  |
| Time + Condition + Group |  | 0.053 |  | 0.003 |  | 0.058 |  | 189.614 |  | 17.635 |  |
| Time + Condition + Time  x  Condition |  | 0.053 |  | 0.003 |  | 0.047 |  | 232.265 |  | 7.118 |  |
| Time + Condition + Group + Condition  x  Group |  | 0.053 |  | 5.514e -4 |  | 0.010 |  | 1107.491 |  | 11.747 |  |
| Time + Condition + Group + Time  x  Group |  | 0.053 |  | 4.889e -4 |  | 0.009 |  | 1249.182 |  | 2.475 |  |
| Time + Condition + Group + Time  x  Condition |  | 0.053 |  | 4.724e -4 |  | 0.009 |  | 1292.629 |  | 2.131 |  |
| Time + Condition + Group + Time  x  Group + Condition  x  Group |  | 0.053 |  | 1.004e -4 |  | 0.002 |  | 6080.135 |  | 6.529 |  |
| Time + Condition + Group + Time  x  Condition + Time  x  Group |  | 0.053 |  | 9.595e -5 |  | 0.002 |  | 6364.547 |  | 4.482 |  |
| Time + Condition + Group + Time  x  Condition + Condition  x  Group |  | 0.053 |  | 9.351e -5 |  | 0.002 |  | 6530.527 |  | 4.045 |  |
| Time + Condition + Group + Time  x  Condition + Time  x  Group + Condition  x  Group + Time  x  Condition  x  Group |  | 0.053 |  | 3.263e -5 |  | 5.874e -4 |  | 18715.595 |  | 34.039 |  |
| Time + Condition + Group + Time  x  Condition + Time  x  Group + Condition  x  Group |  | 0.053 |  | 2.006e -5 |  | 3.610e -4 |  | 30449.656 |  | 5.661 |  |
|  | | | | | | | | | | | |
| Note:  All models include subject | | | | | | | | | | | |

| **Table 10. Results of Bayesian repeated measures ANOVA for the P1 effect at P3 electrode** | | | | | | | | | | | |
| --- | --- | --- | --- | --- | --- | --- | --- | --- | --- | --- | --- |
| **Models** | | **P(M)** | | **P(M\|data)** | | **BF _M_** | | **BF _01_** | | **error %** | |
| Null model (incl. subject) |  | 0.053 |  | 0.493 |  | 17.470 |  | 1.000 |  |  |  |
| Time |  | 0.053 |  | 0.154 |  | 3.269 |  | 3.204 |  | 0.989 |  |
| Group |  | 0.053 |  | 0.135 |  | 2.820 |  | 3.636 |  | 1.968 |  |
| Condition |  | 0.053 |  | 0.076 |  | 1.470 |  | 6.523 |  | 1.936 |  |
| Time + Group |  | 0.053 |  | 0.044 |  | 0.823 |  | 11.269 |  | 2.581 |  |
| Time + Condition |  | 0.053 |  | 0.024 |  | 0.443 |  | 20.494 |  | 3.236 |  |
| Condition + Group |  | 0.053 |  | 0.020 |  | 0.362 |  | 25.011 |  | 1.313 |  |
| Time + Condition + Time  x  Condition |  | 0.053 |  | 0.020 |  | 0.360 |  | 25.141 |  | 2.694 |  |
| Time + Group + Time  x  Group |  | 0.053 |  | 0.009 |  | 0.168 |  | 53.109 |  | 2.976 |  |
| Time + Condition + Group |  | 0.053 |  | 0.006 |  | 0.115 |  | 77.429 |  | 2.993 |  |
| Condition + Group + Condition  x  Group |  | 0.053 |  | 0.006 |  | 0.113 |  | 78.632 |  | 1.905 |  |
| Time + Condition + Group + Time  x  Condition |  | 0.053 |  | 0.006 |  | 0.112 |  | 79.630 |  | 7.385 |  |
| Time + Condition + Group + Condition  x  Group |  | 0.053 |  | 0.002 |  | 0.042 |  | 212.494 |  | 12.756 |  |
| Time + Condition + Group + Time  x  Condition + Condition  x  Group |  | 0.053 |  | 0.002 |  | 0.033 |  | 269.342 |  | 4.154 |  |
| Time + Condition + Group + Time  x  Group |  | 0.053 |  | 0.001 |  | 0.024 |  | 365.437 |  | 2.788 |  |
| Time + Condition + Group + Time  x  Condition + Time  x  Group |  | 0.053 |  | 0.001 |  | 0.022 |  | 404.946 |  | 5.326 |  |
| Time + Condition + Group + Time  x  Group + Condition  x  Group |  | 0.053 |  | 4.431e -4 |  | 0.008 |  | 1111.490 |  | 6.465 |  |
| Time + Condition + Group + Time  x  Condition + Time  x  Group + Condition  x  Group |  | 0.053 |  | 4.275e -4 |  | 0.008 |  | 1152.120 |  | 9.216 |  |
| Time + Condition + Group + Time  x  Condition + Time  x  Group + Condition  x  Group + Time  x  Condition  x  Group |  | 0.053 |  | 1.209e -4 |  | 0.002 |  | 4074.973 |  | 6.449 |  |
|  | | | | | | | | | | | |
| Note:  All models include subject | | | | | | | | | | | |

| **Table 11. Results of Bayesian repeated measures ANOVA for the P1 effect at P4 electrode** | | | | | | | | | | | |
| --- | --- | --- | --- | --- | --- | --- | --- | --- | --- | --- | --- |
| **Models** | | **P(M)** | | **P(M\|data)** | | **BF _M_** | | **BF _01_** | | **error %** | |
| Null model (incl. subject) |  | 0.053 |  | 0.398 |  | 11.885 |  | 1.000 |  |  |  |
| Time |  | 0.053 |  | 0.155 |  | 3.313 |  | 2.558 |  | 0.905 |  |
| Group |  | 0.053 |  | 0.137 |  | 2.857 |  | 2.904 |  | 0.925 |  |
| Condition |  | 0.053 |  | 0.071 |  | 1.385 |  | 5.564 |  | 1.546 |  |
| Time + Group |  | 0.053 |  | 0.054 |  | 1.019 |  | 7.425 |  | 1.374 |  |
| Condition + Group + Condition  x  Group |  | 0.053 |  | 0.037 |  | 0.693 |  | 10.724 |  | 1.863 |  |
| Time + Condition |  | 0.053 |  | 0.029 |  | 0.545 |  | 13.534 |  | 3.986 |  |
| Condition + Group |  | 0.053 |  | 0.024 |  | 0.441 |  | 16.615 |  | 1.456 |  |
| Time + Condition + Group + Condition  x  Group |  | 0.053 |  | 0.019 |  | 0.344 |  | 21.195 |  | 13.623 |  |
| Time + Group + Time  x  Group |  | 0.053 |  | 0.018 |  | 0.334 |  | 21.802 |  | 1.787 |  |
| Time + Condition + Time  x  Condition |  | 0.053 |  | 0.016 |  | 0.291 |  | 24.993 |  | 2.287 |  |
| Time + Condition + Group + Time  x  Condition + Condition  x  Group |  | 0.053 |  | 0.012 |  | 0.211 |  | 34.383 |  | 14.326 |  |
| Time + Condition + Group |  | 0.053 |  | 0.010 |  | 0.173 |  | 41.769 |  | 2.065 |  |
| Time + Condition + Group + Time  x  Condition |  | 0.053 |  | 0.006 |  | 0.105 |  | 68.435 |  | 5.755 |  |
| Time + Condition + Group + Time  x  Group + Condition  x  Group |  | 0.053 |  | 0.005 |  | 0.095 |  | 75.942 |  | 4.271 |  |
| Time + Condition + Group + Time  x  Condition + Time  x  Group + Condition  x  Group |  | 0.053 |  | 0.003 |  | 0.061 |  | 117.736 |  | 9.060 |  |
| Time + Condition + Group + Time  x  Group |  | 0.053 |  | 0.003 |  | 0.060 |  | 120.551 |  | 6.269 |  |
| Time + Condition + Group + Time  x  Condition + Time  x  Group |  | 0.053 |  | 0.002 |  | 0.034 |  | 213.274 |  | 2.943 |  |
| Time + Condition + Group + Time  x  Condition + Time  x  Group + Condition  x  Group + Time  x  Condition  x  Group |  | 0.053 |  | 8.717e -4 |  | 0.016 |  | 456.214 |  | 5.251 |  |
|  | | | | | | | | | | | |
| Note:  All models include subject | | | | | | | | | | | |

| **Table 12. Results of Bayesian repeated measures ANOVA models for the N1 effect at P3 electrode** | | | | | | | | | | | |
| --- | --- | --- | --- | --- | --- | --- | --- | --- | --- | --- | --- |
| **Models** | | **P(M)** | | **P(M\|data)** | | **BF _M_** | | **BF _01_** | | **error %** | |
| Null model (incl. subject) |  | 0.053 |  | 0.438 |  | 14.053 |  | 1.000 |  |  |  |
| Time |  | 0.053 |  | 0.185 |  | 4.097 |  | 2.365 |  | 1.246 |  |
| Group |  | 0.053 |  | 0.142 |  | 2.976 |  | 3.090 |  | 0.881 |  |
| Condition |  | 0.053 |  | 0.070 |  | 1.350 |  | 6.283 |  | 2.018 |  |
| Time + Group |  | 0.053 |  | 0.060 |  | 1.154 |  | 7.274 |  | 1.638 |  |
| Time + Condition |  | 0.053 |  | 0.031 |  | 0.585 |  | 13.932 |  | 10.193 |  |
| Condition + Group |  | 0.053 |  | 0.021 |  | 0.393 |  | 20.536 |  | 1.498 |  |
| Time + Condition + Time  x  Condition |  | 0.053 |  | 0.014 |  | 0.262 |  | 30.520 |  | 5.430 |  |
| Time + Group + Time  x  Group |  | 0.053 |  | 0.012 |  | 0.220 |  | 36.355 |  | 2.111 |  |
| Time + Condition + Group |  | 0.053 |  | 0.009 |  | 0.171 |  | 46.657 |  | 2.576 |  |
| Condition + Group + Condition  x  Group |  | 0.053 |  | 0.006 |  | 0.100 |  | 79.056 |  | 14.883 |  |
| Time + Condition + Group + Time  x  Condition |  | 0.053 |  | 0.004 |  | 0.075 |  | 106.316 |  | 2.092 |  |
| Time + Condition + Group + Time  x  Group |  | 0.053 |  | 0.002 |  | 0.034 |  | 235.356 |  | 2.284 |  |
| Time + Condition + Group + Condition  x  Group |  | 0.053 |  | 0.002 |  | 0.033 |  | 242.908 |  | 2.374 |  |
| Time + Condition + Group + Time  x  Condition + Condition  x  Group |  | 0.053 |  | 8.514e -4 |  | 0.015 |  | 514.968 |  | 3.843 |  |
| Time + Condition + Group + Time  x  Condition + Time  x  Group |  | 0.053 |  | 8.149e -4 |  | 0.015 |  | 538.008 |  | 3.233 |  |
| Time + Condition + Group + Time  x  Group + Condition  x  Group |  | 0.053 |  | 3.871e -4 |  | 0.007 |  | 1132.500 |  | 3.892 |  |
| Time + Condition + Group + Time  x  Condition + Time  x  Group + Condition  x  Group |  | 0.053 |  | 1.547e -4 |  | 0.003 |  | 2834.074 |  | 2.997 |  |
| Time + Condition + Group + Time  x  Condition + Time  x  Group + Condition  x  Group + Time  x  Condition  x  Group |  | 0.053 |  | 7.185e -5 |  | 0.001 |  | 6102.017 |  | 9.141 |  |
|  | | | | | | | | | | | |
| Note:  All models include subject | | | | | | | | | | | |

| **Table 13. Results of Bayesian repeated measures ANOVA models for N1 effect at P4 electrode** | | | | | | | | | | | |
| --- | --- | --- | --- | --- | --- | --- | --- | --- | --- | --- | --- |
| **Models** | | **P(M)** | | **P(M\|data)** | | **BF _M_** | | **BF _01_** | | **error %** | |
| Null model (incl. subject) |  | 0.053 |  | 0.535 |  | 20.749 |  | 1.000 |  |  |  |
| Time |  | 0.053 |  | 0.152 |  | 3.218 |  | 3.531 |  | 1.014 |  |
| Group |  | 0.053 |  | 0.124 |  | 2.541 |  | 4.328 |  | 2.768 |  |
| Condition |  | 0.053 |  | 0.079 |  | 1.549 |  | 6.757 |  | 2.403 |  |
| Time + Group |  | 0.053 |  | 0.035 |  | 0.650 |  | 15.369 |  | 2.055 |  |
| Time + Condition |  | 0.053 |  | 0.021 |  | 0.390 |  | 25.258 |  | 0.966 |  |
| Condition + Group |  | 0.053 |  | 0.018 |  | 0.324 |  | 30.300 |  | 1.604 |  |
| Time + Group + Time  x  Group |  | 0.053 |  | 0.011 |  | 0.201 |  | 48.435 |  | 2.736 |  |
| Time + Condition + Time  x  Condition |  | 0.053 |  | 0.010 |  | 0.178 |  | 54.543 |  | 4.759 |  |
| Time + Condition + Group |  | 0.053 |  | 0.005 |  | 0.091 |  | 106.889 |  | 2.247 |  |
| Condition + Group + Condition  x  Group |  | 0.053 |  | 0.004 |  | 0.067 |  | 145.401 |  | 2.167 |  |
| Time + Condition + Group + Time  x  Condition |  | 0.053 |  | 0.002 |  | 0.038 |  | 251.785 |  | 2.278 |  |
| Time + Condition + Group + Time  x  Group |  | 0.053 |  | 0.002 |  | 0.028 |  | 338.986 |  | 2.121 |  |
| Time + Condition + Group + Condition  x  Group |  | 0.053 |  | 0.001 |  | 0.019 |  | 507.690 |  | 3.447 |  |
| Time + Condition + Group + Time  x  Condition + Time  x  Group |  | 0.053 |  | 7.765e -4 |  | 0.014 |  | 689.614 |  | 8.250 |  |
| Time + Condition + Group + Time  x  Condition + Condition  x  Group |  | 0.053 |  | 5.137e -4 |  | 0.009 |  | 1042.333 |  | 10.301 |  |
| Time + Condition + Group + Time  x  Group + Condition  x  Group |  | 0.053 |  | 3.606e -4 |  | 0.006 |  | 1485.006 |  | 3.744 |  |
| Time + Condition + Group + Time  x  Condition + Time  x  Group + Condition  x  Group |  | 0.053 |  | 1.588e -4 |  | 0.003 |  | 3371.752 |  | 7.461 |  |
| Time + Condition + Group + Time  x  Condition + Time  x  Group + Condition  x  Group + Time  x  Condition  x  Group |  | 0.053 |  | 5.469e -5 |  | 9.845e -4 |  | 9791.179 |  | 27.608 |  |
|  | | | | | | | | | | | |
| Note:  All models include subject | | | | | | | | | | | |

| **Table 14. Results of Bayesian repeated measures ANOVA for LPD effect** | | | | | | | | | | | |
| --- | --- | --- | --- | --- | --- | --- | --- | --- | --- | --- | --- |
| **Models** | | **P(M)** | | **P(M\|data)** | | **BF _M_** | | **BF _01_** | | **error %** | |
| Null model (incl. subject) |  | 0.053 |  | 0.276 |  | 6.856 |  | 1.000 |  |  |  |
| Time |  | 0.053 |  | 0.267 |  | 6.548 |  | 1.034 |  | 1.522 |  |
| Group |  | 0.053 |  | 0.105 |  | 2.112 |  | 2.627 |  | 1.106 |  |
| Time + Group |  | 0.053 |  | 0.105 |  | 2.103 |  | 2.637 |  | 4.805 |  |
| Condition |  | 0.053 |  | 0.061 |  | 1.174 |  | 4.505 |  | 20.022 |  |
| Time + Condition |  | 0.053 |  | 0.047 |  | 0.882 |  | 5.906 |  | 1.550 |  |
| Time + Condition + Time  x  Condition |  | 0.053 |  | 0.032 |  | 0.602 |  | 8.519 |  | 11.631 |  |
| Time + Group + Time  x  Group |  | 0.053 |  | 0.021 |  | 0.379 |  | 13.359 |  | 1.837 |  |
| Condition + Group |  | 0.053 |  | 0.019 |  | 0.355 |  | 14.267 |  | 3.584 |  |
| Time + Condition + Group |  | 0.053 |  | 0.019 |  | 0.345 |  | 14.671 |  | 6.731 |  |
| Time + Condition + Group + Time  x  Condition |  | 0.053 |  | 0.011 |  | 0.207 |  | 24.217 |  | 3.160 |  |
| Time + Condition + Group + Condition  x  Group |  | 0.053 |  | 0.010 |  | 0.184 |  | 27.321 |  | 20.405 |  |
| Time + Condition + Group + Time  x  Condition + Condition  x  Group |  | 0.053 |  | 0.009 |  | 0.160 |  | 31.401 |  | 29.811 |  |
| Condition + Group + Condition  x  Group |  | 0.053 |  | 0.008 |  | 0.143 |  | 35.087 |  | 1.838 |  |
| Time + Condition + Group + Time  x  Group |  | 0.053 |  | 0.004 |  | 0.070 |  | 71.342 |  | 2.852 |  |
| Time + Condition + Group + Time  x  Group + Condition  x  Group |  | 0.053 |  | 0.002 |  | 0.045 |  | 110.366 |  | 36.053 |  |
| Time + Condition + Group + Time  x  Condition + Time  x  Group |  | 0.053 |  | 0.002 |  | 0.044 |  | 113.784 |  | 4.624 |  |
| Time + Condition + Group + Time  x  Condition + Time  x  Group + Condition  x  Group |  | 0.053 |  | 0.001 |  | 0.019 |  | 257.487 |  | 4.349 |  |
| Time + Condition + Group + Time  x  Condition + Time  x  Group + Condition  x  Group + Time  x  Condition  x  Group |  | 0.053 |  | 7.428e -4 |  | 0.013 |  | 371.357 |  | 56.298 |  |
|  | | | | | | | | | | | |
| Note:  All models include subject | | | | | | | | | | | |

| **Table 15. Results of attentional bias and attentional disengagement models based on validity effect** | | | | |  |  |
| --- | --- | --- | --- | --- | --- | --- |
| Variables | Df | F | P | Partial η^2^ | | |
| Attentional Bias (N = 34) |  |  |  |  | | |
| Time | 1 | 0.87 | 0.352 | 0.006 | | |
| Condition | 1 | 1.72 | 0.192 | 0.012 | | |
| Group | 1 | 0.43 | 0.510 | 0.003 | | |
| Time x Condition | 1 | 0.15 | 0.696 | 0.001 | | |
| Time x Group | 1 | 1.28 | 0.259 | 0.009 | | |
| Condition x Group | 1 | 1.24 | 0.266 | 0.009 | | |
| Time x Condition x Group | 1 | 0.38 | 0.535 | 0.002 | | |
| Error | 128 |  |  |  | | |
| Attentional Disengagement (N = 34) |  |  |  |  | | |
| Time | 1 | 1.77 | 0.184 | 0.012 | | |
| Condition | 1 | 4.86 | 0.029* | 0.034 | | |
| Group | 1 | 3.03 | 0.084 | 0.021 | | |
| Time x Condition | 1 | 1.21 | 0.272 | 0.008 | | |
| Time x Group | 1 | 0.08 | 0.768 | <0.001 | | |
| Condition x Group | 1 | 0.39 | 0.531 | 0.002 | | |
| Time x Condition x Group | 1 | 0.28 | 0.596 | 0.002 | | |
| Error | 128 |  |  |  | | |

*Note:* Participants with missing values and erroneous data were excluded from the analyses. Participants who do not show validity effect in the pre-intervention period of both conditions were excluded (validity effect = invalidly cued target > validly cued target).

| **Table 16. Results of EDAN and LDAP models based on validity effect** | | | | | | | | | | | | | | | | |
| --- | --- | --- | --- | --- | --- | --- | --- | --- | --- | --- | --- | --- | --- | --- | --- | --- |
| Variables | | | Df | | | | F | | | | P | | | Partial η^2^ | | |
| EDAN at 190-250 ms. (P3/P4) (N = 32) | | |  | | | |  | | | |  | | |  | | |
| Time | | | 1 | | | | 1.76 | | | | 0.194 | | | 0.056 | | |
| Condition | | | 1 | | | | 3.79 | | | | 0.061 | | | 0.112 | | |
| Group | | | 1 | | | | 2.75 | | | | 0.107 | | | 0.084 | | |
| Time x Condition | | | 1 | | | | 0.86 | | | | 0.361 | | | 0.028 | | |
| Time x Group | | | 1 | | | | 1.41 | | | | 0.244 | | | 0.045 | | |
| Condition x Group | | | 1 | | | | 0.07 | | | | 0.789 | | | 0.002 | | |
| Time x Condition x Group | | | 1 | | | | 0.11 | | | | 0.741 | | | 0.004 | | |
| Error | | | 30 | | | |  | | | |  | | |  | | |
| LDAP at 560-640 ms. (T3/T4) (N = 32) | | |  | | | |  | | | |  | | |  | | |
| Time | | | 1 | | | | 0.10 | | | | 0.749 | | | 0.003 | | |
| Condition | | | 1 | | | | 0.74 | | | | 0.396 | | | 0.024 | | |
| Group | | | 1 | | | | 1.14 | | | | 0.293 | | | 0.037 | | |
| Time x Condition | | | 1 | | | | 0.08 | | | | 0.769 | | | 0.003 | | |
| Time x Group | | | 1 | | | | 3.70 | | | | 0.064 | | | 0.110 | | |
| Condition x Group | | | 1 | | | | 0.14 | | | | 0.707 | | | 0.005 | | |
| Time x Condition x Group | | | 1 | | | | 2.55 | | | | 0.121 | | | 0.078 | | |
| Error | | | 30 | | | |  | | | |  | | |  | | |
| *Note:* Participants with missing values and erroneous data were excluded from the analyses. Participants who do not show validity effect in the pre intervention period of both conditions were excluded (validity effect = invalidly cued target > validly cued target). | | | | | | | | | | | | | | | | |
| \| **Table 17. Results of EDAN and LDAP models in the neutral condition based on validity effect** \| \| \| \| \| \| \| --- \| --- \| --- \| --- \| --- \| --- \| \| Variables \| \| Df \| F \| P \| Partial η^2^ \| \| EDAN at 240-280 ms. (P3/P4) (N = 32) \|  \| \|  \|  \|  \| \| Time \| \| 1 \| 2.11 \| 0.156 \| 0.066 \| \| Group \| \| 1 \| 2.50 \| 0.124 \| 0.012 \| \| Time x Group \| \| 1 \| 0.37 \| 0.543 \| 0.012 \| \| Error \| \| 30 \|  \|  \|  \| \| LDAP at 560-640 ms. (T3/T4) (N = 32) \| \|  \|  \|  \|  \| \| Time \| \| 1 \| 0.17 \| 0.683 \| 0.006 \| \| Group \| \| 1 \| 0.36 \| 0.550 \| 0.012 \| \| Time x Group \| \| 1 \| <0.01 \| 0.954 \| <0.001 \| \| Error \| \| 30 \|  \|  \|  \|   *Note:* Participants with missing values and erroneous data were excluded from the analyses. Participants who do not show validity effect in the pre-intervention period of both conditions were excluded (validity effect = invalidly cued target > validly cued target).  **Table 18. Results of EDAN and LDAP models in the reward condition based on validity effect** | | | | | | | | | | | | | | |  |  |
| Variables | | | | | Df | | | F | | P | | | Partial η^2^ | |  |  |
| EDAN at 240-280 ms. (P3/P4) (N = 32) | | | |  | | | |  | |  | | |  | |  |  |
| Time | | | | | 1 | | | 0.20 | | 0.651 | | | 0.007 | |  |  |
| Group | | | | | 1 | | | 1.91 | | 0.177 | | | 0.060 | |  |  |
| Time x Group | | | | | 1 | | | 1.61 | | 0.213 | | | 0.051 | |  |  |
| Error | | | | | 30 | | |  | |  | | |  | |  |  |
| LDAP at 560-640 ms. (T3/T4) (N = 32) | | | | |  | | |  | |  | | |  | |  |  |
| Time | | | | | 1 | | | <0.01 | | 0.973 | | | <0.001 | |  |  |
| Group | | | | | 1 | | | 1.09 | | 0.303 | | | 0.035 | |  |  |
| Time x Group | | | | | 1 | | | 6.70 | | 0.015* | | | 0.183 | |  |  |
| Error | | | | | 30 | | |  | |  | | |  | |  |  |
| *Note:* Participants with missing values and erroneous data were excluded from the analyses. Participants who do not show validity effect in the pre-intervention period of both conditions were excluded (validity effect = invalidly cued target > validly cued target). The analyses were conducted for the reward condition. | | | | | | | | | | | | | | |  |  |
| **Table 19: Results of P1 models based on validity effect** | | | | | | | | | | | | | | | |  |
| Variables |  |  | Df | | | F | | | p | | | Partial η^2^ | | | |  |
| P1 at 100-137 ms. (P3) (N = 32) | | |  | | |  | | |  | | |  | | | |  |
| Time |  |  | 1 | | | 0.23 | | | 0.880 | | | 0.001 | | | |  |
| Condition | |  | 1 | | | <0.01 | | | 0.941 | | | <0.001 | | | |  |
| Group |  |  | 1 | | | 0.06 | | | 0.796 | | | 0.002 | | | |  |
| Time x Condition | |  | 1 | | | 0.20 | | | 0.651 | | | 0.007 | | | |  |
| Time x Group | |  | 1 | | | 3.49 | | | 0.075 | | | 0.102 | | | |  |
| Condition x Group | |  | 1 | | | 0.53 | | | 0.472 | | | 0.017 | | | |  |
| Time x Condition x Group | | | 1 | | | 1.52 | | | 0.227 | | | 0.048 | | | |  |
| Error |  |  | 30 | | |  | | |  | | |  | | | |  |
| P1 at 100-137 ms. (P4) (N = 32) | | |  | | |  | | |  | | |  | | | |  |
| Time |  |  | 1 | | | 0.27 | | | 0.603 | | | 0.009 | | | |  |
| Condition | |  | 1 | | | <0.01 | | | 0.996 | | | <0.001 | | | |  |
| Group |  |  | 1 | | | 1.80 | | | 0.189 | | | 0.057 | | | |  |
| Time x Condition | |  | 1 | | | 0.09 | | | 0.758 | | | 0.003 | | | |  |
| Time x Group | |  | 1 | | | 0.40 | | | 0.532 | | | 0.013 | | | |  |
| Condition x Group | |  | 1 | | | 2.72 | | | 0.110 | | | 0.083 | | | |  |
| Time x Condition x Group | | | 1 | | | 0.32 | | | 0.572 | | | 0.011 | | | |  |
| Error |  |  | 30 | | |  | | |  | | |  | | | |  |
| *Note:* Participants with missing values and erroneous data were excluded from the analyses. Participants who do not show validity effect in the pre-intervention period of both conditions were excluded (validity effect = invalidly cued target > validly cued target). | | | | | | | | | | | | | | | |  |
| **Table 20: Results of N1 models based on validity effect** | | | | | | | | | | | | | | | |  |
| Variables |  |  | Df | | | F | | | p | | | Partial η^2^ | | | |  |
| N1 at 141-188 ms. (P3) (N = 32) | | |  | | |  | | |  | | |  | | | |  |
| Time |  |  | 1 | | | 0.72 | | | 0.401 | | | 0.024 | | | |  |
| Condition | |  | 1 | | | 0.34 | | | 0.854 | | | 0.001 | | | |  |
| Group |  |  | 1 | | | 2.43 | | | 0.129 | | | 0.075 | | | |  |
| Time x Condition | |  | 1 | | | 0.02 | | | 0.882 | | | 0.001 | | | |  |
| Time x Group | |  | 1 | | | 1.21 | | | 0.279 | | | 0.039 | | | |  |
| Condition x Group | |  | 1 | | | 0.10 | | | 0.753 | | | 0.003 | | | |  |
| Time x Condition x Group | | | 1 | | | 0.19 | | | 0.665 | | | 0.006 | | | |  |
| Error |  |  | 30 | | |  | | |  | | |  | | | |  |
| N1 at 141-188 ms. (P4) (N = 32) | | |  | | |  | | |  | | |  | | | |  |
| Time |  |  | 1 | | | 0.80 | | | 0.377 | | | 0.026 | | | |  |
| Condition | |  | 1 | | | 0.17 | | | 0.681 | | | 0.006 | | | |  |
| Group |  |  | 1 | | | 0.84 | | | 0.367 | | | 0.027 | | | |  |
| Time x Condition | |  | 1 | | | 0.77 | | | 0.384 | | | 0.025 | | | |  |
| Time x Group | |  | 1 | | | 0.01 | | | 0.918 | | | <0.001 | | | |  |
| Condition x Group | |  | 1 | | | 0.41 | | | 0.522 | | | 0.014 | | | |  |
| Time x Condition x Group | | | 1 | | | 0.07 | | | 0.780 | | | 0.003 | | | |  |
| Error |  |  | 30 | | |  | | |  | | |  | | | |  |
| *Note:* Participants with missing values and erroneous data were excluded from the analyses. Participants who do not show validity effect in the pre-intervention period of both conditions were excluded (validity effect = invalidly cued target > validly cued target).   \| **Table 21: Results of the LPD model based on validity effect** \| \| \| \| \| \| \| \| \| \| \| \| \| \| --- \| --- \| --- \| --- \| --- \| --- \| --- \| --- \| --- \| --- \| --- \| --- \| --- \| \| Variables \| \|  \| \|  \| Df \| \| F \| \| p \| \| Partial η^2^ \| \| \| LPD at 229-299 ms. (Cz) (N = 32) \| \| \| \| \|  \| \|  \| \|  \| \|  \| \| \| Time \| \|  \| \|  \| 1 \| \| 0.39 \| \| 0.534 \| \| 0.013 \| \| \| Condition \| \| \| \|  \| 1 \| \| 0.16 \| \| 0.691 \| \| 0.005 \| \| \| Group \| \|  \| \|  \| 1 \| \| 0.85 \| \| 0.363 \| \| 0.028 \| \| \| Time x Condition \| \| \| \|  \| 1 \| \| 1.21 \| \| 0.279 \| \| 0.039 \| \| \| Time x Group \| \| \| \|  \| 1 \| \| 3.81 \| \| 0.060 \| \| 0.113 \| \| \| Condition x Group \| \| \| \|  \| 1 \| \| 2.03 \| \| 0.165 \| \| 0.063 \| \| \| Time x Condition x Group \| \| \| \| \| 1 \| \| <0.01 \| \| 0.952 \| \| <0.001 \| \| \| Error \| \|  \| \|  \| 30 \| \|  \| \|  \| \|  \| \| \| *Note:* Participants with missing values and erroneous data were excluded from the analyses. Participants who do not show validity effect in the pre-intervention period of both conditions were excluded (validity effect = invalidly cued target > validly cued target). \| \| \| \| \| \| \| \| \| \| \| \| \| \| **Table 22. Results of P1 models in the reward condition based on validity effect** \| \| \| \| \| \| \| \| \| \| \| \| \| Variables \|  \|  \| Df \| \| \| F \| \| p \| \| Partial η^2^ \| \| \| P1 at 100-137 ms. (P3) (N = 32) \| \| \|  \| \| \|  \| \|  \| \|  \| \| \| Time \|  \|  \| 1 \| \| \| 0.05 \| \| 0.809 \| \| 0.002 \| \| \| Group \|  \|  \| 1 \| \| \| 0.31 \| \| 0.576 \| \| 0.011 \| \| \| Time x Group \| \|  \| 1 \| \| \| 6.30 \| \| 0.018* \| \| 0.174 \| \| \| Error \|  \|  \| 30 \| \| \|  \| \|  \| \|  \| \| \| P1 at 100-137 ms. (P4) (N = 32) \| \| \|  \| \| \|  \| \|  \| \|  \| \| \| Time \|  \|  \| 1 \| \| \| 0.02 \| \| 0.873 \| \| 0.001 \| \| \| Group \|  \|  \| 1 \| \| \| 3.06 \| \| 0.090 \| \| 0.093 \| \| \| Time x Group \| \|  \| 1 \| \| \| 0.61 \| \| 0.441 \| \| 0.020 \| \| \| Error \|  \|  \| 30 \| \| \|  \| \|  \| \|  \| \| \| *Note:* Participants with missing values and erroneous data were excluded from the analyses. Participants who do not show validity effect in the pre-intervention period of both conditions were excluded (validity effect = invalidly cued target > validly cued target). The analyses were conducted for the reward condition. \| \| \| \| \| \| \| \| \| \| \| \| | | | | | | | | | | | | | | | |  |

| **Table 23. Results of N1 models in the reward condition based on validity effect** | | | | | | |
| --- | --- | --- | --- | --- | --- | --- |
| Variables |  |  | Df | F | p | Partial η^2^ |
| N1 at 141-188 ms. (P3) (N = 32) | | |  |  |  |  |
| Time |  |  | 1 | 1.84 | 0.671 | 0.006 |
| Group |  |  | 1 | 1.42 | 0.242 | 0.045 |
| Time x Group | |  | 1 | 1.33 | 0.718 | 0.004 |
| Error |  |  | 30 |  |  |  |
| N1 at 141-188 ms. (P4) (N = 32) | | |  |  |  |  |
| Time |  |  | 1 | <0.01 | 0.977 | <0.001 |
| Group |  |  | 1 | 0.07 | 0.784 | 0.003 |
| Time x Group | |  | 1 | 0.01 | 0.903 | 0.001 |
| Error |  |  | 30 |  |  |  |
| *Note:* Participants with missing values and erroneous data were excluded from the analyses. Participants who do not show validity effect in the pre-intervention period of both conditions were excluded (validity effect = invalidly cued target > validly cued target). The analyses were conducted for the reward condition. | | | | | | |

| **Table 24. Results of LPD models in the reward condition based on validity effect** | | | | | | |
| --- | --- | --- | --- | --- | --- | --- |
| Variables |  |  | Df | F | p | Partial η^2^ |
| LPD at 229-299 ms. (Cz) (N = 32) | | |  |  |  |  |
| Time |  |  | 1 | 0.09 | 0.760 | 0.003 |
| Group |  |  | 1 | 0.01 | 0.898 | 0.001 |
| Time x Group | |  | 1 | 1.57 | 0.219 | 0.050 |
| Error |  |  | 30 |  |  |  |
| *Note:* Participants with missing values and erroneous data were excluded from the analyses. Participants who do not show validity effect in the pre-intervention period of both conditions were excluded (validity effect = invalidly cued target > validly cued target). The analyses were conducted for the reward condition. | | | | | | |
